# Smartphone-Based Physical Activity Telecoaching in Chronic Obstructive Pulmonary Disease: Mixed-Methods Study on Patient Experiences and Lessons for Implementation

**DOI:** 10.2196/mhealth.9774

**Published:** 2018-12-21

**Authors:** Matthias Loeckx, Roberto A Rabinovich, Heleen Demeyer, Zafeiris Louvaris, Rebecca Tanner, Noah Rubio, Anja Frei, Corina De Jong, Elena Gimeno-Santos, Fernanda M Rodrigues, Sara C Buttery, Nicholas S Hopkinson, Gilbert Büsching, Alexandra Strassmann, Ignasi Serra, Ioannis Vogiatzis, Judith Garcia-Aymerich, Michael I Polkey, Thierry Troosters

**Affiliations:** 1 Department of Rehabilitation Sciences KU Leuven Leuven Belgium; 2 Respiratory Division University Hospitals Leuven Leuven Belgium; 3 Department of Physiotherapy LUNEX International University of Health, Exercise and Sports Differdange Luxembourg; 4 ELEGI Colt Laboratory, Centre for Inflammation Research The Queen's Medical Research Institute University of Edinburgh Edinburgh United Kingdom; 5 ISGlobal Barcelona Spain; 6 Faculty of Physical Education and Sports Sciences National and Kapodistrian University of Athens Athens Greece; 7 National Institute for Health Research Respiratory Biomedical Research Unit Royal Brompton and Harefield National Health Services Foundation Trust and Imperial College London United Kingdom; 8 Epidemiology, Biostatistics and Prevention Institute University of Zurich Zurich Switzerland; 9 Groningen Research Institute for Asthma and Chronic Obstructive Pulmonary Disease-Primary Care Department of General Practice and Elderly Care University of Groningen, University Medical Center Groningen Groningen Netherlands; 10 CIBER Epidemiología y Salud Pública Barcelona Spain; 11 Universitat Pompeu Fabra Barcelona Spain; 12 Department of Sport, Exercise and Rehabilitation Faculty of Health and Life Sciences Northumbria University Newcastle-upon-Tyne United Kingdom

**Keywords:** physical activity, COPD, telemedicine, smartphone, patient adherence, patient satisfaction, outcome and process assessment (health care)

## Abstract

**Background:**

Telecoaching approaches can enhance physical activity (PA) in patients with chronic obstructive pulmonary disease (COPD). However, their effectiveness is likely to be influenced by intervention-specific characteristics.

**Objective:**

This study aimed to assess the acceptability, actual usage, and feasibility of a complex PA telecoaching intervention from both patient and coach perspectives and link these to the effectiveness of the intervention.

**Methods:**

We conducted a mixed-methods study based on the completers of the intervention group (N=159) included in an (effective) 12-week PA telecoaching intervention. This semiautomated telecoaching intervention consisted of a step counter and a smartphone app. Data from a project-tailored questionnaire (quantitative data) were combined with data from patient interviews and a coach focus group (qualitative data) to investigate patient and coach acceptability, actual usage, and feasibility of the intervention. The degree of actual usage of the smartphone and step counter was also derived from app data. Both actual usage and perception of feasibility were linked to objectively measured change in PA.

**Results:**

The intervention was well accepted and perceived as feasible by all coaches present in the focus group as well by patients, with 89.3% (142/159) of patients indicating that they enjoyed taking part. Only a minority of patients (8.2%; 13/159) reported that they found it difficult to use the smartphone. Actual usage of the step counter was excellent, with patients wearing it for a median (25th-75th percentiles) of 6.3 (5.8-6.8) days per week, which did not change over time (*P*=.98). The smartphone interface was used less frequently and actual usage of all daily tasks decreased significantly over time (*P*<.001). Patients needing more contact time had a smaller increase in PA, with mean (SD) of +193 (SD 2375) steps per day, +907 (SD 2306) steps per day, and +1489 (SD 2310) steps per day in high, medium, and low contact time groups, respectively; *P* for-trend=.01. The overall actual usage of the different components of the intervention was not associated with change in step count in the total group (*P*=.63).

**Conclusions:**

The 12-week semiautomated PA telecoaching intervention was well accepted and feasible for patients with COPD and their coaches. The actual usage of the step counter was excellent, whereas actual usage of the smartphone tasks was lower and decreased over time. Patients who required more contact experienced less PA benefits.

**Trial Registration:**

ClinicalTrials.gov NCT02158065; http://clinicaltrials.gov/ct2/show/NCT02158065 (Archived by WebCite at http://www.webcitation.org/73bsaudy9)

## Introduction

### Background

Reduction in physical activity (PA) is a major feature of chronic obstructive pulmonary disease (COPD), occurring both as a consequence of disease and driving worse outcomes in the condition [1]. PA coaching has been recommended as a nonpharmacological treatment strategy for patients with COPD across all stages of the disease [2]. Telecoaching, where support is provided to achieve effective behavior change by use of electronic communication strategies [3], has received increasing attention in the recent years. It offers the possibility of coaching patients from a distance in an automated or semiautomated way, thereby reducing the burden of face-to-face interactions for patients and health care providers. The latter type of intervention is an example of a complex intervention, which consists of several interacting components [4]. This interaction between multiple components complicates the implementation of such interventions [4]. Therefore, process evaluations have been proposed by the UK Medical Research Council [5], which offer the possibility to investigate how the intervention was delivered (ie, why the intervention worked or did not work) in addition to whether it was effective or not. This is of crucial importance to health technology assessment bodies as it provides information on which components of an intervention were effective or noneffective and how the intervention can be improved and replicated in different settings and patient groups [4,5]. Process evaluation can also be of great value in evaluating PA telecoaching interventions, which have been shown to be effective in enhancing PA in some studies [6-8] but not in others [9]. In a recent multicenter PA telecoaching trial (MrPAPP), which had a positive outcome [6], a large variability in the effect of the intervention was noticed. Patients with better functional exercise capacity (ie, 6-minute walking distance [6MWD] ≥450 meters), fewer symptoms (ie, modified Medical Research Council [mMRC] dyspnea scale <2), those in Global Initiative for Chronic Obstructive Lung Disease (GOLD) quadrants A-B improved their PA to a greater extent [6]. In addition to these patient characteristics, intervention-specific characteristics and the way patients cope with the intervention might also have contributed to the success of the intervention.

### Objectives

In this paper, 3 concepts, which are often assessed as part of a process evaluation, have been investigated: (1) acceptability, (2) actual usage, and (3) feasibility of the intervention from both a patient and a coach perspective. In addition, we aimed to investigate their association (ie, actual usage and feasibility) with the effectiveness of the intervention.

First, acceptability is a key concept in the development, evaluation, and the implementation of complex interventions and can have significant impact on the intervention’s effectiveness [10]. It has been defined as “a multi-faceted construct that reflects the extent to which people delivering or receiving a health care intervention consider it to be appropriate, based on anticipated or experienced cognitive and emotional responses to the intervention” [10]. A potentially effective intervention might not reach its potential due to poor acceptability to patients or health care providers [10].

Second, the actual usage of the intervention by patients and health care providers forms an important part of the delivery of PA telecoaching interventions. Actual usage was assessed as the degree to which patients used the components of the interventions as it was designed [11]. It is often confused with the term *adherence* [12]. The latter term requires a rationale for the minimum intended use of the components of the intervention. As there is no established minimum usage of such PA telecoaching interventions, we used the term *actual usage*, with the assumption that the more usage, the better [12]. Although the actual usage of step counters is known to be relatively good in short-term coaching trials involving patients with COPD [7,13,14], actual usage of smartphone apps in coaching trials has been less intensively studied.

Third, the implementation of this intervention also depends on whether it was considered to be feasible by patients as well by the coaches. Feasibility is defined as “the extent to which a new treatment, or an innovation, can be successfully used or carried out within a given agency or setting” [15,16]. The coach feasibility of the PA telecoaching program in this paper has already been partly assessed in the main paper of the MrPAPP trial, which reported that coaches contacted patients for a total duration of 50 min throughout the trial [6]. However, qualitative data on the perceived feasibility of both patient and coach are lacking.

Finally, the direct association between both coach feasibility (as assessed by contact time) and actual usage by patients with the effectiveness of the intervention was investigated. The latter insights could lead to improved design and implementation of PA telecoaching interventions in the future as well as optimized selection of patients.

## Methods

### Ethics Approval

This study was approved by the local ethics committee at each center (Commissie medische ethiek van de universitaire ziekenhuizen KU Leuven [Leuven, S-55919]; Medische ethische toetsingscommissie universitair medisch centrum Groningen [Groningen, Metc 2013.362]; RES Committee London—South East [London and Edinburgh, 13/LO/1660]; Scientific Council of the ‘Sotiria’ General Hospital for Chest Diseases (Athens, 27852/7-10-13); Kantonale Ethikkommission Zürich, and Ethikkommission Nordwest- und Zentralschweiz [Zurich, KEK-ZH-Nr. 2013-0469 and EKNZ2014-192, respectively]).

### Study Population and Design

A convergent mixed-methods research design using quantitative and qualitative data was applied to evaluate the acceptability, actual usage, and feasibility of a PA telecoaching intervention. Both qualitative and quantitative data on the intervention were separately collected and analyzed. Later, these findings were compared for data triangulation, which allowed a more comprehensive understanding of the intervention [17-19].

This trial forms part of a 12-week, multicenter randomized controlled trial (1:1 randomization) conducted by the PROactive consortium [6]. The trial consisted of 3 visits—a screening visit (V1), a randomization visit (V2) 1 to 2 weeks later, and a final visit (V3) 12 weeks post randomization. In total, 171 patients were allocated to the intervention group in 6 centers across Europe (Leuven, Belgium; Athens, Greece; London and Edinburgh, United Kingdom; Zurich, Switzerland; and Groningen, The Netherlands) between June and December 2014, from which 159 patients completed the trial and were considered for the present analyses. More information on the study population and design has already been published elsewhere [6]. All patients provided informed consent before any data collection.

### Physical Activity Telecoaching Intervention

**Figure 1:**
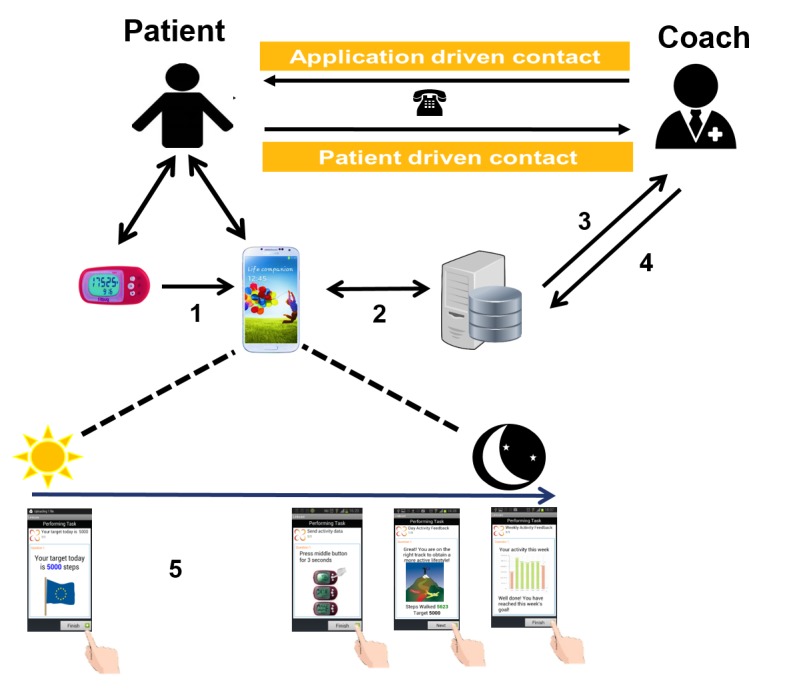
Overview of the intervention; 1=sending of “steps data” to smartphone (through Bluetooth); 2=data sent to central database; 3=coach is able to access database; 4=coach is able to manually adjust goals, 5=accessing & closing the different tasks on the smartphone app (automated messages); i.e., (from left to right); morning goal, send activity in the evening, daily feedback (from Monday to Saturday) and weekly feedback (only on Sunday) tasks.

Patients in the intervention group [6] received a multicomponent PA telecoaching intervention, consisting of a step counter and a smartphone app (Samsung Galaxy S4 mini; android version 4.4.2), in addition to usual care. Furthermore, patients in the intervention group received an exercise instruction booklet for home use and a one-to-one interview with a coach discussing motivation, barriers, favorite activities, and strategies to become more active. The exercise instruction booklet contained 3 different sessions of upper limb and lower limb stretching as well as balance and strengthening exercises with a standardized amount of sets and repetitions (see Multimedia Appendix 1). Patients were asked to wear the step counter (Fitbug air) during waking hours and to interact with the project-tailored smartphone app on a daily basis. They were instructed to access and review automated tasks that appeared on the smartphone’s display and to press the *close* *box* on the screen thereafter (ie, completion of a task). An audio reminder was provided for patients to send their step data at 8 pm to their smartphone (through Bluetooth) by pressing a single button of the step counter. The app provided patients with daily activity goals in the morning, which were set for 1 week. The patients’ goal was adjusted according to their PA performance in the previous week and to their willingness to increase their goal. Goals were calculated based on the mean and median of the 4 most active days of the previous week. If the mean value was higher than the weekly goal (ie, patients reaching the goal), the patients had the opportunity to (1) not change or (2) increase their median goal by 500 steps through a *yes* or *no* option displayed on the app. If the mean of the 4 most active days of the previous week was lower (ie, patients not reaching their goal) and the median was more than 500 steps below the goal, the goal was reduced to the median of the 4 most active days+500 steps. In other cases, the goal remained the same. Coaches were asked to contact the patients (ie, tasks of the coaches) in case patients (1) did not send their step count data for 3 consecutive days, (2) did not reach their target for 2 consecutive weeks, (3) reached the target but they were not willing to increase for 2 consecutive weeks, and (4) were not adherent with wearing the step counter for 2 consecutive weeks. More details on when coaches were instructed to contact the patients (ie, flagging system) are published elsewhere [6]. Daily and weekly encouraging feedback messages were displayed on the smartphone using both text and pictograms (see Multimedia Appendix 2; slide 7). Throughout the whole intervention period, coaches could access patient data through their app-linked Web accounts to monitor patients’ performed PA and their actual usage of the intervention (PROactive Linkcare app, Barcelona, Spain; see Multimedia Appendix 2). The use of the intervention was completely free of charge for all patients. No major bug fixes or changes to the intervention were made throughout the trial. A detailed overview of how the intervention works can be found in Figure 1.

### Outcomes

#### Acceptability

Acceptability was assessed through quantitative data (a project-tailored questionnaire [20 items, Multimedia Appendix 3]) and qualitative data collection (patient interview [4 open questions, Multimedia Appendix 4] and a coach focus group [Multimedia Appendix 5]).

During the final visit of the study (V3), patients were asked to fill in a 20-min self-administered, project-tailored, multiple-choice questionnaire on their experiences with the intervention and the usefulness of its components on a 10-point Likert scale (Multimedia Appendix 3). Each center collected and anonymized answers from all their patients into an Excel file, which was sent to 1 investigator (HD). HD pooled all data together into 1 Excel file, which was then used for analysis.

Patient interviews were conducted by local PA coaches in each center at V3. Each coach was informed and trained on how to conduct the interview during an investigator’s meeting before the start of the trial. Interviewers from each center were asked to transcribe the answers of the patients to the discussion guide questions and forward them (anonymized) to one researcher (ML) who collected all quotes into 1 Excel file for analysis. In this pooled Excel file, each line represented the verbatim answer of each participant on a question with a number code and a letter representing, respectively, the patient’s ID and the question of the discussion guide.

After completion of the trial, an audiotaped focus group was organized to capture the intervention experience from the perspective of the coaches. Local PA coaches with a diverse background (ie, medical doctor [RAR], physiotherapist [ML, HD], exercise physiologist [ZL], biomedical scientist [MS], and psychologist [AF]; n=6), and 2 experienced physiotherapists who were involved in the development of the intervention (n=2; EGS and Ane Arbillaga-Etxarri (AAE) from the center in Barcelona [IS GLOBAL]) discussed the feasibility, appreciation, possible future adaptations, time investment, and actual usage of the different components of the intervention (Multimedia Appendix 5). A total of 2 PA coaches (ML and HD) facilitated the focus group.

#### Actual Usage

Actual usage of the intervention by patients was assessed objectively through the smartphone app log. A database was derived directly from the smartphone app. This included information about completion of the app tasks and step counter data on a day-by-day basis. Actual usage of the step counter was defined based on the presence of step count data (ie, ≥70 steps for that day). Self-reported actual usage of performing home exercise and the times patients looked at their step counter were assessed subjectively in the project-tailored questionnaire.

Actual usage by the coaches was assessed based on the closure of tasks in the app-linked Web accounts and discussed during the coach focus group.

#### Feasibility

Coach feasibility was already partly assessed in the main paper of the MrPAPP trial in terms of number of contacts and total amount of contact time between coaches and patients (quantitative data) [6]. As a secondary analysis, the evolution in efficiency of coaches, as measured by contact time throughout the study recruitment period, was assessed. In addition, coach perception of the feasibility of the intervention was also covered in the coach focus group (qualitative data). Intervention feasibility from the patient perspective was evaluated through the project-tailored questionnaire (quantitative data) and patient interviews (qualitative data).

#### Association of Actual Usage and Feasibility With the Effectiveness of the Intervention

Both actual usage by patients and coach feasibility (ie, contact time) with the intervention were separately linked to the effectiveness of the intervention. This effectiveness was assessed as the change in numbers of steps per day after 12 weeks, measured by the Actigraph GT3x (ACT, Actigraph LLC Pensacola, FL). The latter is a triaxial accelerometer validated for use in patients with COPD [20,21]. Further details on the PA assessment methodology and its validity criteria can be found elsewhere [6].

### Statistical Analysis

All statistical analyses were performed with Statistical Analysis Software version 9.4 (SAS Institute, Cary, NC). Continuous variables were expressed as means with SD (normal distribution) or as medians (25th-75th percentiles [P25-P75]; skewed distribution), unless stated otherwise. Categorical variables were expressed as proportions and percentages. The level of significance was set at .05 for all statistical tests. The analyses were based on patients in the intervention group who completed the 12-week intervention (N=159).

Data from the project-tailored questionnaire were scored as categorical variables and reported as frequencies and percentages (ie, number of patients indicating each answer), except for the usefulness ratings of the components, which were expressed as median (P25-P75).

For analysis of the interview data, two researchers (HD and FMR) independently performed thematic analysis on the Excel file containing the verbatim transcriptions of the interview data [22] according to the 6-step framework as proposed by Braun and Clarkes [23]:

1. HD and FMR read the data multiple times and descriptively noted down their initial ideas of what is in the data and what is interesting about them.
2. HD and FMR independently generated an initial list of codes from the data and put the data systematically under certain headings.
3. Afterwards, they searched for reoccurring themes, which began to emerge from these codes to focus their analysis on a broader level.
4. HD and FMR refined and defined their themes taken into account the overall message of the analysis. Themes and subthemes were organized and ranked into categories.
5. HD and FMR came together for group discussion to find an agreement on defining the themes and subthemes, which led to the development of a (final) codebook.
6. Afterwards, one researcher (ML) applied the final codebook to all verbatim transcripts. After iterative group discussions, data were synthesized and representative example quotes were extracted to illustrate findings and were labeled by a unique participant’s code together with the category of contact time and actual usage score of that participant.

The thematic analysis was conducted inductively (ie, themes emerged from the data, hence without predetermined coding frame) in Excel, without the use of specialized analytic software. Further details on the methodological aspects of the latter analyses have been added to the COnsolidated criteria for REporting Qualitative research (COREQ) checklist (see Multimedia Appendix 6).

During the focus group, 1 PA coach (ML) wrote a consensus summary. A total of 2 PA coaches (HD and MS) independently reviewed the consensus summary based on the audio recording. Additional information that was considered as relevant was independently added by both coaches (HD and MS). Only minor interpretation disagreements occurred between the 2 PA coaches, which were discussed together with a third PA coach (ML). Later, a summary of the focus group was sent for revision to all PA coaches, including those who could not be present at the focus group. A consensus quote on the future implementation of this PA telecoaching intervention was formulated.

Actual usage was compared according to age (<65 vs ≥65 years, Mann-Whitney *U* test), gender (male vs female, Mann-Whitney *U* test), and over time in the trial (week 2-3 vs week 11-12, Wilcoxon signed-rank sum test). Actual usage of the step counter was expressed as the percentage of patients who wore the step counter for at least 90% of the days in the study. Actual usage of the different smartphone tasks was expressed as median (P25-P75).

In the larger centers (inclusion of at least 20 patients), the contact time with the first 10 patients was compared with the others (Mann-Whitney *U* test) to assess possible learning effect of the coaches.

**Figure 2:**
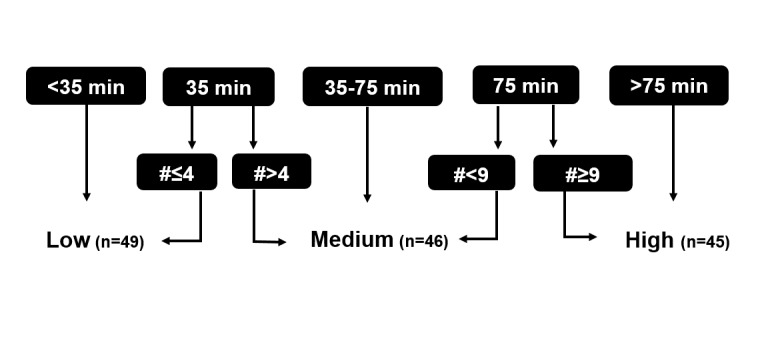
Division (into 3 groups) of patients based on total duration and number of contacts between patients and coach. Min=minutes; #=number of contacts; n=number of patients in each group.

We attempted to create 3 equally balanced groups (low, medium, and high) of total contact time (Figure 2) and of an overall score of actual usage. This overall actual usage score was calculated by summing up each actual usage component (actual usage of all tasks and wearing the step counter) as a percentage of their recommended frequency. The 3 groups were compared (via analysis of variance test or Kruskal-Wallis test) to characterize those who required a lot of contact time and those who did not and those who had high actual usage of the intervention and those who did not. As a sensitivity analysis for the latter tertiles approach, we also analyzed contact time and actual usage score as continuous variables. The methodology used for the latter sensitivity analysis can be found in Multimedia Appendix 7.

To analyze the association between (1) the actual usage by patients of different components of the intervention and coach feasibility (ie, contact time) and (2) the effectiveness of the intervention, 2 separate generalized linear model analyses were used in completers with valid PA data (88.1% [140/159] of the completers sample). Change in PA was used as the outcome and contact time and actual usage as the class variables, respectively. Due to their possible influence on the intervention effect, baseline exercise capacity (6MWD), symptom score (mMRC-scale), forced expiratory volume in 1 second (FEV_1_) % predicted, and the number of acute exacerbations in the previous 12 months were considered as possible (continuous) covariates of the association [6]. Details on sensitivity analyses for the latter tertiles approach (with contact time and actual usage scores as continuous variables) can be found in Multimedia Appendix 7. Finally, we hypothesized high contact time in the first 4 weeks to be an early sign of absence of response to the intervention. To that end, we calculated the likelihood of achieving the minimal important difference (MID) improvement of 1000 steps per day [24] in patients with a low (≤30 min) and high (>30 min) contact time in the first 4 weeks of the trial (as a possible early predictor for treatment failure).

## Results

### Study Population

Baseline characteristics of the 159 completers are outlined in Table 1. Information on the full study population (including further details about dropouts and the occurrence of adverse events) has been detailed elsewhere [6].

### Outcomes

#### Acceptability

Overall, the PA telecoaching intervention was well received by the patients as 89.3% (142/159) indicated that they “enjoyed taking part in the intervention.” Furthermore, the majority of the patients (59.1%, 94/159) claimed that the intervention coached them “a lot” toward enhancing their PA. Approximately half of the patients (47.2%, 75/159) experienced the proposed weekly increases in step counts as “reasonable,” whereas 37.7% (60/159) and 10.1% (16/159) of the patients experienced these increases as “a little bit too high” and “much too high,” respectively.

**Figure 3:**
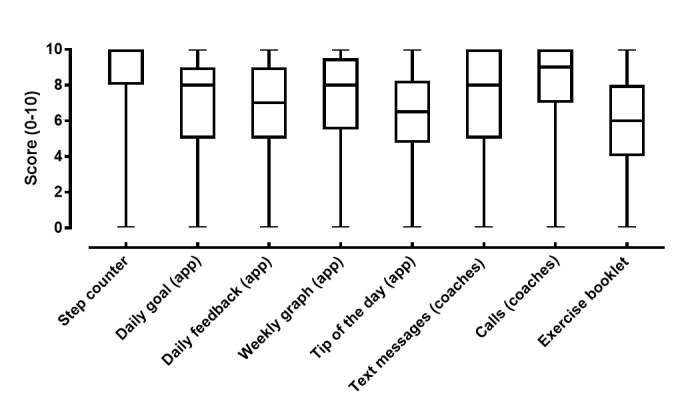
Boxplots depicting the usefulness score (0-10 Likert scale) of the different parts of the intervention from the patients’ perspective. “app” between brackets represents messages displayed on the smartphone app.

Patients rated the usefulness of the step counter (median [P25-P75]; 10 [8-10]) and the telephone contacts with the coach in case of problems (9 [7-10]) as the most crucial parts of the intervention (see Figure 3). The display of a daily (educational) activity tip in the evening (6.5 [5-8]) and the booklet for home exercises (6 [4-8]) were rated as less useful.

When patients were asked to name the most important part of the intervention, 76.1% (121/159) of patients did choose the step counter as the most important part with 93.1% (148/159) of all patients willing to continue using the step counter in the future. In total, 45.9% (73/159) of all patients were willing to continue using the full intervention, with only 8.2% (13/159) of all patients reported to experience working with the smartphone as difficult.

**Table 1:** Baseline characteristics of the completers of the trial.

Variables	Intervention completers (n=159)
Age in years, mean (SD)	66 (8)
Gender (male), n (%)	89 (64)
BMI^a^ (kg/m^2^), mean (SD)	26.9 (5.3)
FEV_1_^b^ predicted (%), mean (SD)	53.9 (19.9)
6MWD^c^ (m), mean (SD)	442 (107)
6MWD predicted (%), mean (SD)	70.3 (16.5)
CAT^d^ score, mean (SD)	13 (8)
QF^e^ (kg), mean (SD)	31.5 (10.9)
PA^f^ (steps per day), median (P25-P75)^g^	4272 (2783-5768)

^a^BMI: body mass index.

^b^FEV_1_: forced expiratory volume in 1 second.

^c^6MWD: 6-minute walking distance; 6MWD was missing in 2 patients.

^d^CAT: chronic obstructive pulmonary disease (COPD) assessment test.

^e^QF: quadriceps force; QF was not measured in 2 centers and QF was missing in 27 patients.

^f^PA: physical activity; valid PA measurements were present in 140 patients.

^g^25th and 75th percentiles (P25-P75).

In total, 91.2% of patients (145/159 of the completers sample) took part in the semistructured interviews at V3. Themes and subthemes that were derived from the verbatim responses of patients to the interview are presented in Textbox 1. Moreover, 2 major topics can be distinguished from the interview data: technical aspects and aspects related to the content of the intervention (see Textbox 1). Illustrative quotes, which support findings from the thematic analysis, are provided in Multimedia Appendix 8. Further information on the interview process, participants, and the interviewers can be found in the COREQ-checklist (see Multimedia Appendix 6).

**Findings of the thematic analysis of the interview data are categorized under (1) technical aspects and (2) aspects related to the content of the intervention.**

**Technical aspects:**

Themes of (1) positive experiences and (2) issues or problems emerged from the data.

1. Positive experiences
  - No technical problems: A large portion of patients stated not to have encountered technical issues with any of the components of the intervention.
  - Working with app: The ease of use with the different components of the intervention was highlighted by patients. Furthermore, patients who had less a priori experience with managing a smartphone device expressed that the learning process of working with this device was smooth.
2. Issues or problems
  - Help from others: Few patients needed more than a familiarization period before they were able to feel confident about working with the smartphone and its app. Help from both the study team (through phone calls or face-to-face contacts) and from their relatives was considered essential when experiencing problems.
  - Speed of interaction with the app: Some patients felt the speed of the app was slow and perceived the interaction with it as time consuming. Especially, the transfer of step data onto the phone in the evening was delayed for several minutes.
  - App problems: Some patients reported during the interview that working with the app was often hindered (eg, tasks not opening and not possible to send data). Reasons for these app problems were mostly related to issues with the internet connection or Bluetooth problems.
  - Step counter: A small minority of patients expressed their frustration with the step counter that was not always able to detect all steps they performed. Activities such as slow walking, cycling, and arm movements were not measured accurately.

**Aspects related to the content of the intervention:**

Themes of (1) positive experiences, (2) issues or problems, and (3) outcome emerged from the data.

1. Positive experiences
  - Step counter: The step counter was judged as the essential part of the intervention by several patients because of its simplicity, feedback, and usefulness.
  - Graphs: Another highly rated aspect of the intervention was the graphical feedback displays that patients received based on the achievement of their goals. According to the patients, it was an interesting and excellent way of motivating them.
  - Nice experience: In general, the intervention was considered as motivating to a large majority of patients across the different centers. Patients claimed it was a fun and interesting experience that helped them toward being more active and feeling better and fitter.
  - Being monitored: One of the most important motivational reasons according to patients to become more active was the feeling of being monitored. Knowing that the coaches were following them up gave them an external motivational cue to be physically active.
  - Family participation: Next to the help from the coaches, patients’ relatives often played an important supportive and stimulating role throughout the intervention. Close relatives of patients (mostly spouses) also bought a step counter to join their wife or husband throughout their coaching.
2. Issues or problems
  - Goals: One of the most important issues was the increase in the step count goal, which was often too high for patients. This caused some frustration among patients as it was perceived as demotivating to have too high goals and not being able to reach them.
  - Variations: As the intervention was used for a period of 12 weeks, the component of variation in the content of the app was deemed as important according to the patients. Some patients reported that because of the lack of variation, their actual usage of the intervention (in particular with the opening of the messages on the smartphone) lowered. The morning messages with the goal patients needed to achieve were repeated every day of that week and required more variation according to the patients.
  - Barriers: One of the major drawbacks of the intervention according to patients was that it did not take into account several barriers with which they were confronted. When a patient experienced an acute exacerbation, his or her goal was not adjusted immediately. Weather factors were not taken into account within the app. Furthermore, patients regretted that there was no option for them to make the intervention aware that they had other priorities (eg, holidays or days when they needed to watch their grandchildren).
  - Motivational issues: A few patients did not find the app interesting and did not like working with it.
3. Outcomes
  - New routine: Patients stated that the intervention and the goals resulted in the adoption of new lifestyle routines to be more physically active. They hoped to continue with these more active lifestyles after the intervention finished.

All coaches present at the focus group considered the intervention to be a useful addition to standard care in patients with COPD. The coaches rated the step counter as very useful, mainly attributed to the direct feedback it provided and its ease of use. Technical problems with the smartphone interface intermittently occurred (eg, Bluetooth connection or requests for automatic updates). In addition, coaches reported that a minority of patients felt the smartphone app lacked variation. Considering future long-term use, coaches proposed a more individualized technical training based on individual patient needs (eg, more extensive in patients with difficulties and those needing more contact time). Finally, the coaches regretted that the home exercises did not result in higher step counts and lacked variation, which might explain the low use of the home exercise booklet by patients.

#### Actual Usage

Almost 60% (59.7%, 95/159) of patients wore the step counter for more than 90% of the days they were included in the coaching program, representing a median (P25-P75) of 6.3 (5.8-6.8) days per week with no difference over time within the trial (*P*=.98). Actual usage of the different smartphone app tasks is outlined in Table 2. Actual usage decreased significantly over time for all tasks (*P*<.001 for all) except for the weekly feedback task (*P*=.14). More specifically, actual usage of the daily goal, sending activity, and daily feedback tasks decreased from, respectively, 5 (3-7), 5 (2.5-6), and 3 (1-5) days per week at the start of the intervention to 4 (1.5-6.5), 3.5 (0.5-6.0), and 2 (0-4.5) days per week at the end of the trial (*P*<.001 for all). The actual usage did not differ between younger and older patients or between male and female patients (Multimedia Appendix 9).

In terms of self-reported actual usage, a large majority of the patients (76.7%, 122/159) stated that they looked *several times per day* at their step counter. Only 22.0% (35/159) of patients claimed to perform their home exercise *at least on a daily basis* and one-third stated they had *never* performed these exercises.

Coaches performed 1053 out of the 1161 contacts that appeared on the platform; however, no details on the time of solving the tasks were available.

#### Feasibility

**Figure 4:**
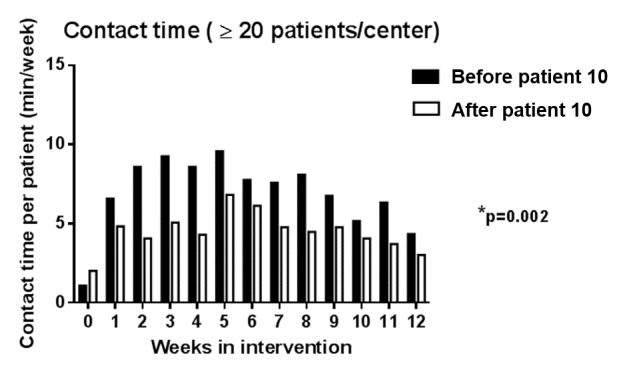
Contact time throughout the intervention (only including centers with more than 20 patients). The black bars represent the mean contact time (in min per week) per patient from the first 10 patients that were recruited in each center. White bars represent the mean contact time (in min per week) per patient from the patients that were recruited at a later stage. P value indicates difference between the total cumulated contact time over the 12 weeks between patients recruited in early stage versus later stage.

Feasibility from the perspective of the patients was good as a large proportion of patients reported that the smartphone intervention was not too much of a burden to work with when they were asked how they had experienced the technical aspects of the intervention. Coaches spent significantly more time (*P*=.002) interacting with the first 10 of their patients compared with the ones who were recruited at a later stage in their center (see Figure 4). These findings were confirmed when the arbitrarily chosen cutoff point of comparing the first 10 patients was changed with the first 8 or 12 patients.

All PA coaches present in the focus group reached consensus that a follow-up of approximately 25 to 30 patients simultaneously for 1 coach would be feasible. It was felt to be beneficial to have 1 coordinating center to discuss day-to-day problems in patient management on a case-by-case approach.

**Table 2:** Overview of the different components of the intervention. Definition of actual usage of the different components of the intervention of all completers (n=159 patients) and the minimum and maximum values one can achieve in terms of actual usage were reported when applicable. Actual usage and possible minimum-maximum are expressed as median (P25-P75) days per week for the step counter and the daily tasks on the app. Weekly feedback is expressed as median (P25-P75) percent of weeks in the intervention.

Components of the intervention		Actual usage		
		Definition of actual usage	Median (p25-p75)^a^	Possible minimum-maximum
One-to-one interview with coach discussing motivation, barriers, favorite activities, and strategies to become more active		N/A^b^	N/A	N/A
Step counter (Fitbug Air; days per week)		A day with ≥70 steps recorded	6.3 (5.8-6.8)	0-7
**A project-tailored smartphone coaching app (Linkcare, Barcelona ES) with different tasks**				
	Send activity data task (days per week)	Patient closes task	4.1 (2.4-5.6)	0-7
	Looking to the daily goal task (days per week)	Patient closes task	4.1 (2.1-5.9)	0-7
	Looking at the daily feedback task (days per week)	Patient closes task	2.2 (0.7-4.1)	0-6
	Looking at the weekly feedback task (% of weeks in the intervention)	Patient closes task	55 (29-78)	0-100
A booklet containing home exercises		N/A	N/A	N/A
Weekly group text messages with activity proposals sent by the coach		N/A	N/A	N/A
Contact with the coaches, which was triggered in the case of nonactual usage with wearing the step counter, failure to transmit data, or failure to progress		N/A	N/A	N/A

^a^25th and 75th percentiles (P25-P75).

^b^N/A: not applicable.

#### Association of Actual Usage and Feasibility With the Effectiveness of the Intervention

**Figure 5:**
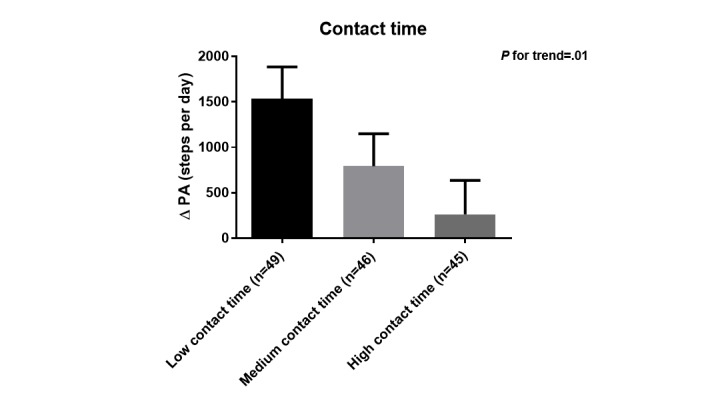
Change in physical activity (PA; mean [SE]) across groups of patients according to total contact time; adjusted for age, baseline functional exercise capacity, baseline forced expiratory volume in 1 second, baseline symptom score and number of acute exacerbations in the previous 12 months. *P* value (*P* for trend) indicates difference in intervention effect between patients divided based on total contact time, after adjusting for the covariates. Data are based on Actigraph measurements and include 140 patients. Unadjusted scores were mean(SD) +1489 (SD 2310) steps per day, +907 (SD 2306) steps per day and +193 (SD 2375) steps per day in low, medium and high contact time groups, respectively.

Patients in the low (n=49), medium (n=46), and high (n=45) contact time group had a median (P25-P75) total contact time of 25 (10-30), 50 (40-60), and 140 (105-185) min, respectively. Patients who had more contact time with the coaches during the time of the study, had more severe airflow obstruction, tended to have a lower functional exercise capacity (Table 3) and had a significant smaller increase in PA, also after adjusting for covariates (age, baseline FEV_1_ [%predicted], baseline 6MWD, baseline mMRC-score, and the number of acute exacerbations in the last 12 months; P-for-trend=.01; Figure 5). The latter findings were confirmed when contact time was treated as a continuous variable (see Multimedia Appendix 7).

**Figure 6:**
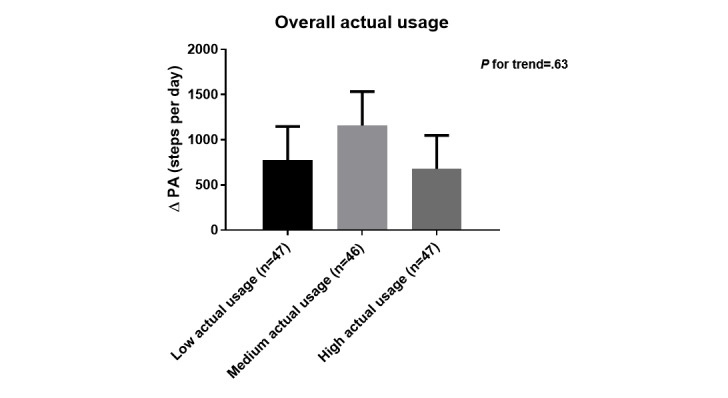
Change in physical activity (PA; mean [SE] across groups of patients according to overall actual usage score; adjusted for age, baseline functional exercise capacity, baseline forced expiratory volume in 1 second, baseline symptom score and number of acute exacerbations in the previous 12 months. *P* value (*P* for trend) indicates difference in intervention effect between patients divided based on the total actual usage score, after adjusting for the covariates. Data are based on Actigraph measurements and include 140 patients. Unadjusted scores were mean(SD) +777 (SD 2767) steps per day, +1159 (SD 2720) steps per day and +679 (SD 2075) steps per day in low, medium and high actual usage groups, respectively.

When groups were divided in 3 according to their overall actual usage score, neither patient characteristics nor effectiveness were different (see Table 4 and Figure 6). The latter findings were confirmed when actual usage score was treated as a continuous variable (sensitivity analyses in Multimedia Appendix 7).

Logistic univariate regression analysis revealed that patients with a low contact time (≤30 min; n=103) after 4 weeks were 3.58 times more likely of achieving the MID improvement of 1000 steps per day (95% CI 1.88-6.82; *P*<.001) compared with patients with more contact time.

**Table 3:** Patient baseline characteristics according to the total contact time (only including patients with valid PA measurement; n=140); data are expressed as mean (SD) unless stated otherwise. P value indicates differences between the 3 contact time groups.

Variables	Low contact time (n=49)	Medium contact time (n=46)	High contact time (n=45)	*P* value
Age in years, mean (SD)	65 (7)	65 (10)	68 (6)	.16
Gender (male), n (%)	28 (57)	34 (74)	27 (60)	.20
BMI^a^ (kg/m^2^), mean (SD)	27.8 (5.3)	26.1 (4.4)	27.0 (6.4)	.35
FEV_1_^b^ predicted percentage, mean (SD)	59.5 (22.6)	54.1 (16.5)	49.1 (20.5)^j^	.04
6MWD^c^ (m), mean (SD)	444 (100)	459 (101)	411 (113)	.09
6MWD predicted percentage, mean (SD)	71.5 (14.5)	71.2 (15.0)	67.4 (19.6)	.29
CAT^d^ score, median (p25-p75)^e^	10 (6-17)	13 (7-19)	16 (10-21)	.11
QF^f^ (kg), mean (SD)	33.1 (13.2)	31.2 (10.0)	29.2 (10.5)	.33
PA^g^ (steps per day), median (p25-p75)	4542 (3387-5587)	4377 (3016-6723)	3186 (2375-5339)	.15
Contact time first 4 weeks in minutes, median (p25-p75)	0 (0-5)^h^	10 (5-20)^i^	50 (20-85)^j^	.005

^a^BMI: body mass index.

^b^FEV_1_: forced expiratory volume in 1 second.

^c^6MWD: 6-minute walking distance; 6MWD was missing in 2 patients.

^d^CAT: chronic obstructive pulmonary disease (COPD) assessment test.

^e^25th and 75th percentiles (P25-P75).

^f^QF: quadriceps force; QF was not measured in 2 centers and QF was missing in 27 patients.

^g^PA: physical activity.

^h^Indicates statistical significance (*P*<.05) between low versus medium contact time groups.

^i^Indicates statistical significance (*P*<.05) between medium versus high contact time groups.

^j^Indicates statistical significance (*P*<.05) between low versus high contact time groups.

**Table 4:** Patient characteristics according to the total actual usage score (3 groups only including patients with valid physical activity measurement by actigraph, n=140); data are expressed as mean (SD) unless stated otherwise. P value indicates differences between the 3 actual usage groups.

Variables	Low actual usage, <47% of usage (n=47)	Medium actual usage, 47% to 75% of usage (n=46)	High actual usage, >75% of usage (n=47)	*P* value
Age in years, mean (SD)	66 (8)	66 (9)	65 (8)	.76
Gender (male), n (%)	31 (66)	29 (63)	29 (62)	.91
BMI^a^ in kg per m^2^, mean (SD)	27.5 (5.3)	27.6 (6.5)	26.0 (4.3)	.34
FEV_1_^b^ predicted percentage, mean (SD)	54.4 (20.3)	55.2 (19.5)	53.5 (21.6)	.92
6MWD^c^ (m), mean (SD)	431 (106)	432 (105)	454 (107)	.50
6MWD predicted percentage, mean (SD)	69 (17)	69 (17)	72 (16)	.61
CAT^d^ score, median (p25-p75)^e^	14 (7-19)	13 (6-19)	12 (7-21)	.94
QF^f^ (kg), mean (SD)	32.0 (10.8)	30.0 (12.9)	31.1 (9.4)	.73
PA^g^ (steps per day) median (p25-p75)	4369 (2868-5672)	3850 (2380-6108)	4540 (2940-6731)	.49

^a^BMI: body mass index.

^b^FEV_1_: forced expiratory volume in 1 second.

^c^6MWD: 6-minute walking distance; 6MWD was missing in 2 patients.

^d^CAT: chronic obstructive pulmonary disease (COPD) assessment test.

^e^25th and 75th percentiles (P25-P75).

^f^QF: quadriceps force; QF was not measured in 2 centers and QF was missing in 27 patients.

^g^PA: physical activity; valid PA measurements was present in 140 patients.

## Discussion

### Principal Findings

On the basis of the secondary analysis of the MrPAPP PA telecoaching trial in patients with COPD, this mixed-methods research design study shows that the intervention was feasible and well accepted by both patients and coaches. Given the design of the intervention (ie, patients were contacted when PA was not increasing), patients with high contact time with coaches had less PA improvements, suggesting that the high contact time resulted from either difficulty or reluctance to engage in PA. Furthermore, we observed that the overall level of actual usage with the program components in the entire group did not influence the intervention effect.

The intervention had good acceptability for patients who rated their satisfaction in line with previous PA telecoaching research in a mixed COPD and diabetes type-2 population [25]. Higher acceptability scores might result in a higher chance of patients having more actual usage of the intervention. This was the case for the high ratings of the step counter by the patients, which was translated into excellent actual usage of the step counter throughout the trial. These high actual usage scores are in line with previous studies [7,9,13,14]. As the step counter was used as the medium to coach patients in this trial, we chose steps per day a priori as primary outcome of the effectiveness of the intervention, which is in line with the initial trial report of the MrPAPP trial [6]. However, one should note that PA encompasses not only amount (eg, steps per day) but also intensity (eg, time spent in moderate to vigorous PA) and time spent in different postures.

The smartphone app was also well received by patients although to a lesser extent than the step counter. This was associated with a considerably lower actual usage score of patients for the smartphone intervention compared with the step counter. Several factors may explain this relatively lower actual usage. First, a proportion of patients with COPD who owned a smartphone before the study might have caused less fluency with the smartphone (*low smartphone literacy*), leading to technical problems and discouraging smartphone use. Unfortunately, we do not have information on smartphone literacy at baseline. Furthermore, the actual usage rate of the smartphone tasks decreased over time. This was against our expectations, as one would expect that patients who have low smartphone literacy at the start of the trial (mostly those without a smartphone of their own) would increase their actual usage over time as they learn to operate the smartphone better. The latter learning effect was often catalyzed through the help of patient’s relative (eg, [grand] children or spouse) and through the study team as reported by patients during the interviews. Second, findings from the semistructured interview revealed that patients felt the interaction with the app was often hindered due to Bluetooth and internet connection issues. Especially, the process of sending the step count data with the smartphone was perceived to be time consuming. This might have caused frustrations among patients, which could have initiated a decline of actual usage of the smartphone. Third, findings from the focus group and patients interviews revealed that patients felt the content of the smartphone app lacked variation (eg, daily repetition of morning messages with the same weekly goal). It presents another probable reason on why actual usage of the smartphone app was rather low and decreased over the 3 months of the trial. This could perhaps be improved by implementing components of gamification [26].

In literature, mixed results and high heterogeneity are reported on the actual usage with PA coaching Web portals or smartphone apps. During a 4-month, internet-based PA telecoaching program, veterans with COPD logged into the website and uploaded their daily step counts for 5.7 days per month which decreased to 3.0 days per month over a follow-up of 12 months [7,27]. Of note, the Web portal in the latter trial was not intended for daily use with a recommended frequency of 4 log-ins per month. The low degree of actual usage over a longer follow-up time was confirmed by a 9-month home-based pilot study, in which a smartphone-based activity coach was rarely used (only for 29 days throughout the whole trial) [28]. However, no information was provided on the change in actual usage over time in the latter trial [28].

Components of the intervention that were not individually tailored (eg, educational activity tips and home exercise booklet) were rated as less useful. This confirms patients’ self-reported actual usage of the home exercise booklet, which was low and is in line with findings from the focus group, in which PA coaches pointed out that the home exercise booklet was not individualized for each specific patient. This highlights the importance of introducing personalized components within PA telecoaching, which has also been suggested in patients with ischemic heart disease who participated in a mobile health cardiac rehabilitation intervention [29,30].

In line with the patients, the coaches expressed good acceptability of this PA telecoaching program. On future use of the intervention, coaches reached the following consensus:

1. “The goal of such a PA telecoaching intervention should be that patients are able to use this intervention quasi independently indefinitely. Every 6 months patients could come for a follow-up visit, synchronized with other planned health visits to the outpatient clinic.” Interestingly, our data suggest that 3 months of coaching might be enough for patients to reach a plateau in PA increase (see Multimedia appendix 10).

2. “As their PA coach it is our task to provide further follow-up by giving them the step counter and occasional phone calls for follow-up.” Such strategies merit further validation, but the statement strengthens the importance of acceptability, actual usage, and feasibility with long-term PA telecoaching programs in this patient population. In addition to the latter perspectives, the coaches highlighted that it is highly important that the preferences and experiences of the patients with the intervention are assessed and taken into account when looking at future implementation. Therefore, future (long-term) PA telecoaching interventions need to ensure whether enough variation within such apps is introduced in addition to those components deemed as the most essential to patients (ie, step counter and contact with the study team). Furthermore, such interventions need to take the occurrence of acute exacerbations into account and involve patients’ relatives as these can play an important role as social support in being physically active [31], which was supported by the analyses of the interview data. Focusing on introducing new daily PA routines can provide a good starting point for long-term PA improvement according to these interview data.

In terms of coach feasibility, the main paper of the MrPAPP trial revealed that patients were contacted for a median of 50 min throughout the 12 weeks intervention [6]. Translated into socioeconomic terms, this means that coaching 25 patients simultaneously corresponds to approximately 2 hours per week for 1 PA coach. This number might even decrease as the coach accumulates his or her expertise or problem-solving efficiency, resulting in a lower burden.

Literature about the relationship of both actual usage by the patients and coach feasibility (contact time) of the intervention with the change in PA in telecoaching trials is scarce. In this study, the degree of the overall actual usage score (including wearing the step counter and all the app tasks) was not associated with the effectiveness of the intervention. This is in contrast to a 4-week pilot (telecoaching) study which showed a positive relationship between the degree of actual usage of wearing a smartphone-based activity coach and the benefits from the intervention during the first 2 weeks albeit this association disappearing during the third week [13]. Next to actual usage of the intervention by patients, actual usage by coaches is also crucial to how the intervention is delivered. Despite a high degree of actual usage of the PA telecoaching program by patients in the trial by Vorrink et al (ie, 89% of the days used) [32], the program was not able to induce significant improvements in PA [9]. The latter might be partly explained by the lack of feasibility from the part of the coaches. Due to financial reasons and time constraints, there was a low degree of actual usage of the primary care physiotherapists in using the foreseen website to adjust the patients’ PA goals and to send motivating messages to the patients. In our trial, actual usage of the coaches could not be assessed in depth as we did not have information on the exact timing when coaches solved the tasks. The latter could have influenced the effectiveness of the intervention. However, the automated goal calculation and adjustment in our intervention could have partly limited the impact on the effectiveness of the intervention in comparison to the trial of Vorrink et al. This highlights the importance of introducing automated or semiautomated components in such interventions.

In contrast to actual usage, the contact time between the coach and patients was associated with the effectiveness of the intervention, that is, a lower effect in those patients in need of more contact time. These patients were the more severe (ie, they have more severe airflow obstruction and tend to have a lower functional exercise capacity) and are more likely to experience exacerbations and therefore, have more chance of triggering coaching-related and/or health-related contacts with their coach. As contact time remained a significant, negative predictor of the change in PA, independent of the patient characteristics, this may point to the inability of some patients to work with the coaching app. This corroborates with the findings of the qualitative part of the study and should not be ignored as a reason for treatment failure. In clinical practice, we would therefore advocate flexible use of these interventions where patients are diverted to other interventions (eg, more supervised exercise programs such as pulmonary rehabilitation) if contact time accumulates. This is important for stratification in future trials.

### Strengths and Limitations

To the best of our knowledge, this study is the first providing an in-depth analysis of the acceptability, actual usage, and feasibility with a PA telecoaching intervention developed for patients with COPD. Our study is unique as it allows us to investigate these aspects, relating them to physiological characteristics along with the level of response.

The results are based on a combination of quantitative and qualitative research, including information coming from patients as well as from coaches. In addition, the study is performed on the back of a properly powered randomized controlled trial, which was characterized by a comprehensive physiological assessment and objective assessment of PA. Furthermore, this PA telecoaching intervention consists of several behavioral principles (including but not limited to facilitating goal setting, action planning, feedback, and problem solving) which were based on the behavior change technique taxonomy of Michie et al [33]. Nevertheless, some limitations need to be considered.

First, we only included patients that completed the trial. This could have resulted in a selection bias. Coaches might have spent more time in those patients who subsequently dropped out during their intervention period. However, as only 7% (12/171) of patients discontinued, this is unlikely to have had a large impact on the results. Second, no multiple-comparison post hoc corrections were applied in the quantitative data analysis as these analyses should be regarded as exploratory and in need of independent confirmation. These results help to guide future research; however, they may not be taken as a final judgment and should be interpreted with caution due to the latter limitation. Third, only 1 focus group with a limited number of PA coaches was performed. Therefore, data saturation could not have been reached. Another focus group with participants with a broad background and experience would have been of great value for (1) external validity of findings and (2) to ensure data saturation. Nevertheless, coaches were asked during the focus group whether they had additional comments. In addition, a summary of the focus group was sent to the coaches who could not be present at the focus group for completion of the summary. New themes emerged, which allowed for more data capturing. Fourth, we did not specifically assess capabilities or history of patients with managing the smartphone device or their expectations. In hindsight, this might have provided even more detailed information to predict the therapeutic response to the PA telecoaching intervention. Fifth, for the assessment of acceptability of the intervention, we used a project-tailored questionnaire. In literature, several attempts have been made to measure the quality of mobile health apps; however, no measure from a user perspective has been widely accepted [34-36]. Incorporating methodologies as proposed within the human computer interaction research and tools such as the mobile app rating scale (MARS) and uMARS (user version) tools (which were not available at the time of trial initialization) would have strengthened the development and validity of the acceptability assessments in this paper [37,38]. Nevertheless, the findings of our project-tailored questionnaire still provide interesting insights into the acceptability with these kinds of interventions. Sixth, as proposed by the Medical Research Council, a process evaluation incorporates 3 themes (ie, implementation, mechanisms of impact, and context) [5]. The concepts of *implementation* and *mechanisms of impact* are largely covered in this paper by the assessments of actual usage, feasibility, and acceptability as well by their association with the effectiveness of the intervention. However, we were not able to evaluate the *context* theme (ie, how external factors had an impact on our intervention) in depth in this study. Seventh, as the cutoffs for making tertiles for contact time and actual usage score were driven by the data collected in this trial, they should not be regarded as clinically important cutoff points despite the wide range of contact time and actual usage scores presented in this paper. Finally, future research should investigate whether a feature for social interactions among peers might further lower the burden on health care providers. Such peer support has also been integrated as a catalyst for behavior change in the taxonomy of Michie et al given that privacy of patients is not breached [26,33].

### Clinical Importance

In line with general findings of the present behavioral modification program [6], this paper shows that PA telecoaching is not an intervention to which all patients respond, but it is feasible and well received by the vast majority of patients. The number of smartphone users is increasing worldwide [39]. Given that it requires only modest health care resources and is relatively less time-consuming compared with one-to-one PA counseling, PA telecoaching does have opportunities for future implementation. Furthermore, the use of an electronic communication strategy might lower the burden on both clinicians and patients as we found a relatively low contact time of 50 min over 3 months of coaching. Moreover, it offers the possibility of coaching people from a distance [3]. The theoretical framework and proven effectiveness of this intervention also provides opportunities for its use in other elderly populations who are in need of being coached toward a more active lifestyle. In addition, findings of this paper provide possible guidance for the selection of patients that will benefit the most from these types of interventions. Patients with very limited exercise capacity, more symptoms, GOLD quadrants C or D, and/or a high amount of contact time during the first 4 weeks of the program are less likely to improve [6]. In these patients, further coaching input may be futile and other more intensive face-to-face interventions should be considered.

### Conclusions

This 12-week PA telecoaching intervention was well accepted and feasible for both patients with COPD and their coaches. Actual usage of the step counter was excellent, whereas actual usage of the smartphone tasks was lower and decreased over time. Overall actual usage was not associated with the effect of the intervention. The step counter and direct contact with the coach were perceived as the most useful components of the intervention by the patients. Patients with more need for contact had more severe airflow obstruction, tended to have more severely limited exercise capacity, and experienced less PA benefits. Alternative strategies (including more face-to-face contacts and offering pulmonary rehabilitation programs) might be more effective in these patients.

## Appendix

Multimedia Appendix 1 Home exercise booklet.

Multimedia Appendix 2 Linkcare application platform.

Multimedia Appendix 3 Project-tailored patient satisfaction form.

Multimedia Appendix 4 Patient interview (discussion guide).

Multimedia Appendix 5 Focus group (coaches).

Multimedia Appendix 6 COREQ checklist.

Multimedia Appendix 7 Sensitivity analyses: contact time & actual usage scores as continuous variables.

Multimedia Appendix 8 Illustrative quotes interviews.

Multimedia Appendix 9 Actual usage of the different intervention tasks according to gender and age.

Multimedia Appendix 10 Overview of (mean ± SE) steps performed per week by all patients (step counter data).

## Abbreviations

COPD: chronic obstructive pulmonary disease
COREQ: COnsolidated criteria for REporting Qualitative research
FEV_1_: forced expiratory volume in 1 second
GOLD: Global Initiative for Chronic Obstructive Lung Disease
MARS: mobile app rating scale
MID: minimal important difference
MrPAPP: multicenter physical activity telecoaching trial
mMRC: modified Medical Research Council
PA: physical activity
6MWD: 6-minute walking distance
