# Supplementary material for: Smartphone-Based Physical Activity Telecoaching in Chronic Obstructive Pulmonary Disease: Mixed-Methods Study on Patient Experiences and Lessons for Implementation
Source: JMIR Mhealth Uhealth. 2018 Dec 21;6(12):e200. doi: 10.2196/mhealth.9774 (PMC6320438; doi:10.2196/mhealth.9774)
Supplement: Multimedia Appendix 1 [file mhealth_v6i12e200_app1.pdf]

Home Exercises

Level 1

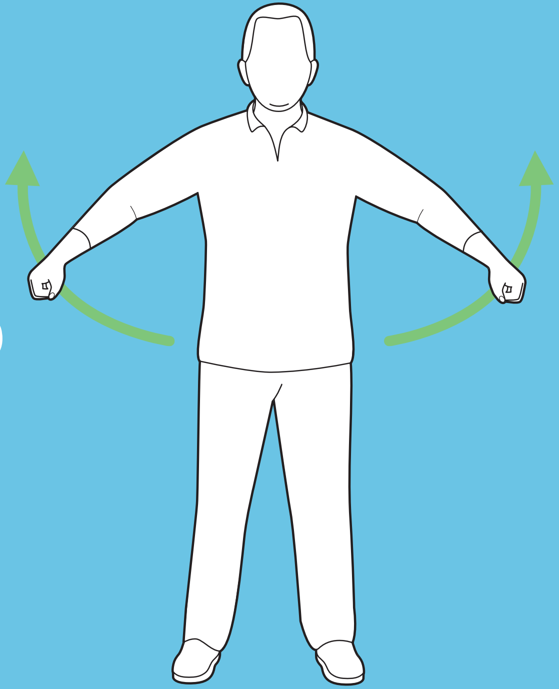

Home exercises

This booklet is part of your health base activity programme.

For each exercise session you can choose from sessions A, B or C.

We hope you enjoy these exercises!

Table of contents

Session A

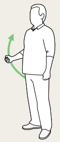

1 - Elbow bends p 1

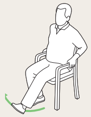

2 - Leg extension p 3

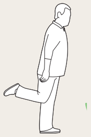

3 - Balance walk p 5

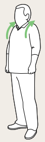

4 - Shoulder shrug p 7

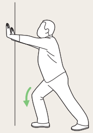

5 - Weight on front foot p 9

Session B

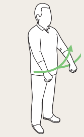
1 - Front arm raise p 11

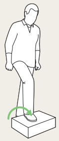
2 - Step up p 13

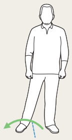
3 - Side step p 15

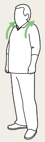
4 - Shoulder shrug p 17

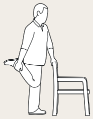
5 - Foot grab p 19

Session C

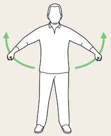
1 - Side arm raise p 21

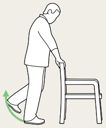
2 - Knee curl p 23

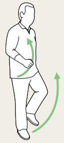
3 - High knee walk p 25

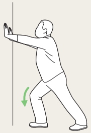
4 - Weight on front foot p 27

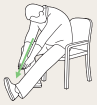
5 - Leg stretch p 29

Session A

Upper extremities

Exercise 1 - Elbow bends

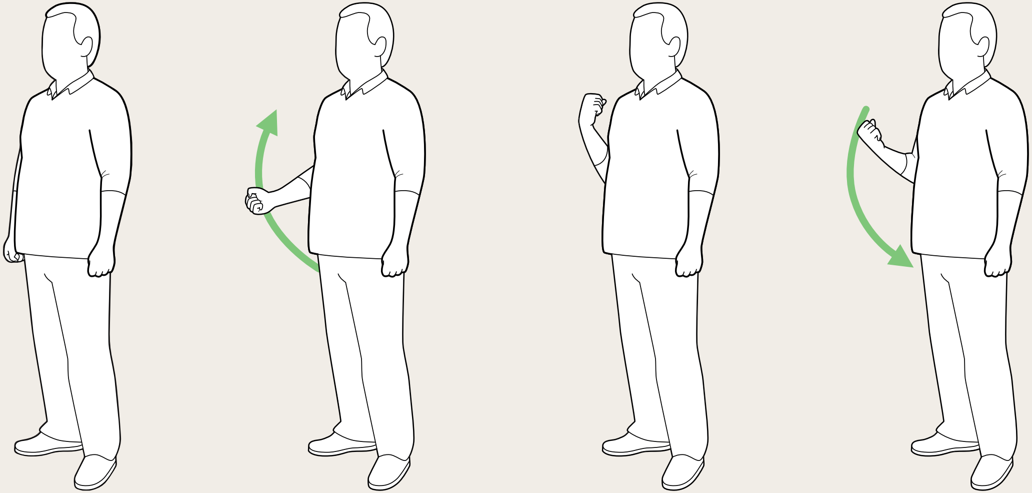

Start by standing with your arms by your side.

Bend your elbow and lift your right hand up to your right shoulder.

Now lower your hand…

3×8

Repetitions

Do 3 sets of 8 repetitions. Rest for 2 minutes after each set.
If you find the exercise too easy, you can do extra repetitions.

When you are finished, turn the page for your next exercise.

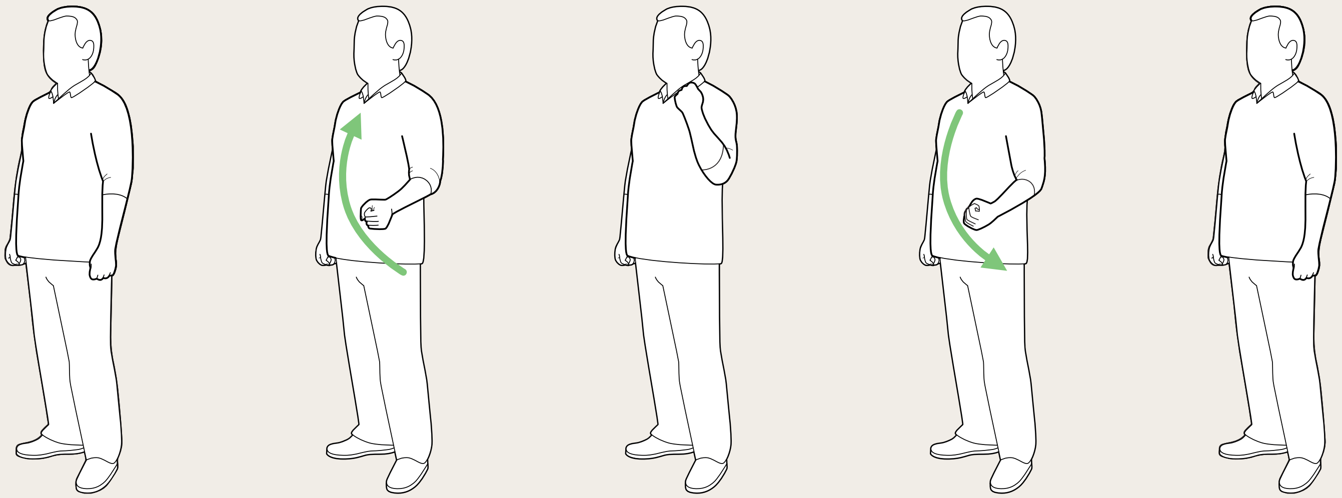

… and straighten your elbow as much as possible.

Now bend your left elbow by lifting your hand up to your left shoulder.

Now lower your hand and straighten your elbow as much as possible.

Session
A

Lower extremities

Exercise 2 - Leg extension

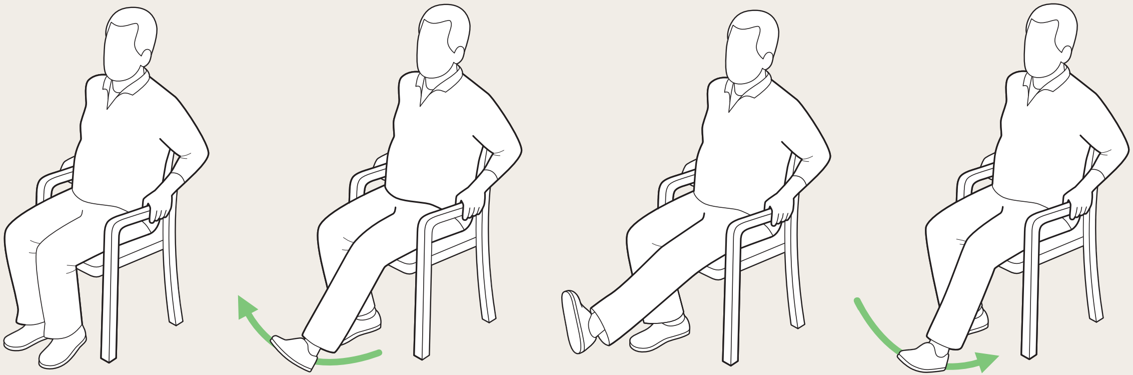

Start by sitting on a chair.

Lift your left foot slowly and straighten your knee. Hold this position and count to 5.

Now lower your leg slowly…

3×8
Repetitions

Do 3 sets of 8 repetitions. Rest for 1 minute after each set.
If you find the exercise too easy, you can do extra repetitions.

When you are finished, turn the page for your next exercise.

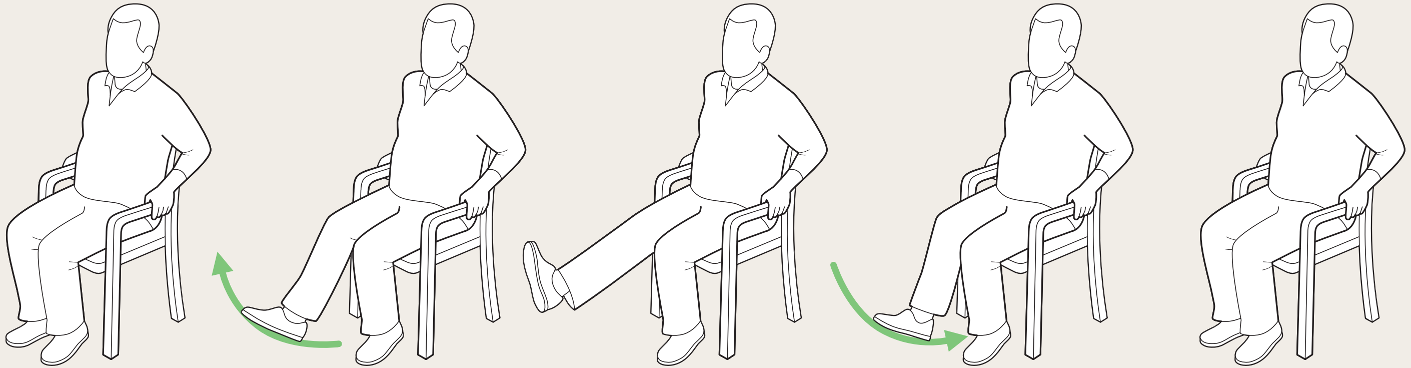

…until your foot is resting on the floor.

Lift your right foot and straighten your knee. Hold this position and count to 5.

Lower your leg slowly until your foot is resting on the floor.

Session
A

Walking exercises

Exercise 3 - Balance walk

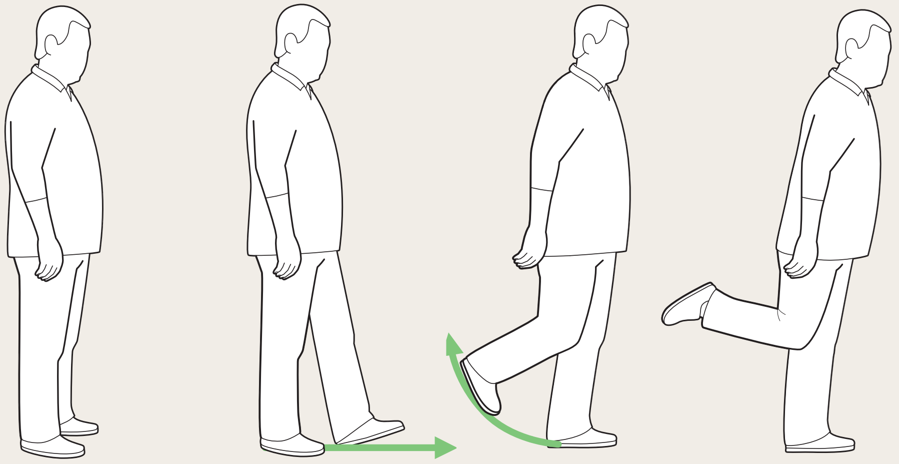

Start by standing with some space in front of you.

Step forward with your left foot.

Now lift your right foot behind you and pause for one second in this position.

3×2 Minutes

Do 3 sets of 2 minutes. Rest for 2 minutes after each set.
If you find the exercise too easy, you can do it for longer.

When you are finished, turn the page for your next exercise.

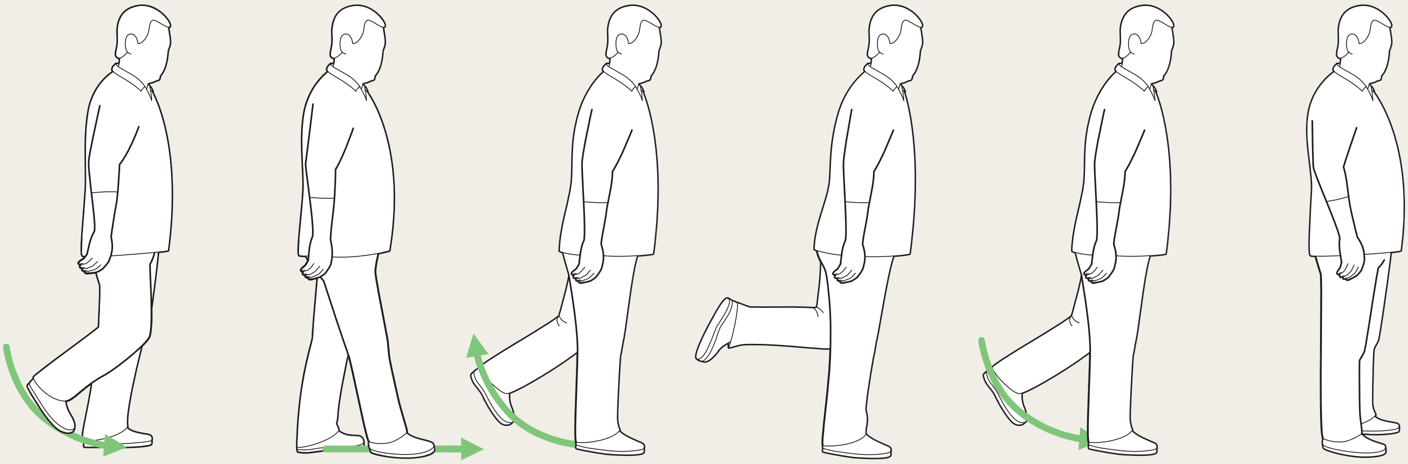

Lower your right foot and take a step forward.

Now lift your left foot behind you and pause for one second in this position.

Lower your left foot and take a step forward.

Session

A

Cooling down

Exercise 4 - Shoulder shrug

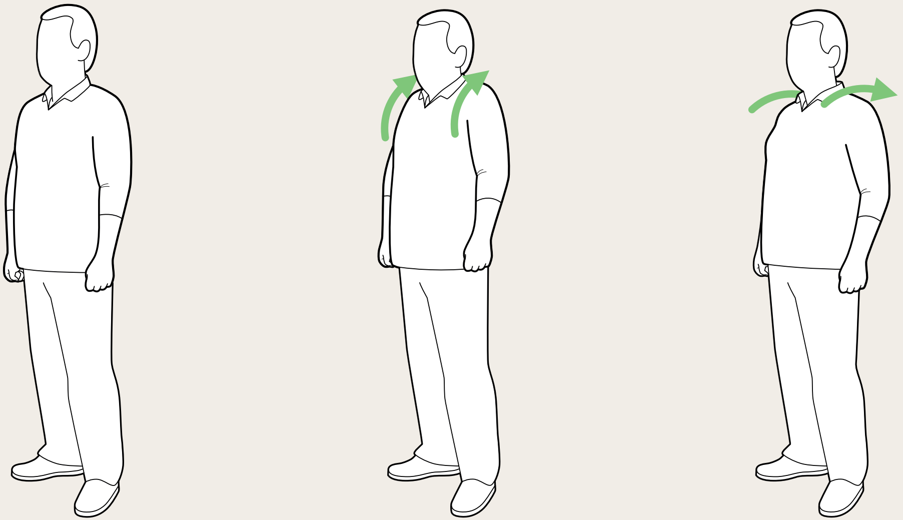

Start by standing with your arms by your side.

Slowly lift your shoulders up and roll them back...

3×5

Repetitions

Do 3 sets of 5 repetitions. Rest for 2 minutes after each set.
If you find the exercise too easy, you can do extra repetitions.

When you are finished, turn the page for your next exercise.

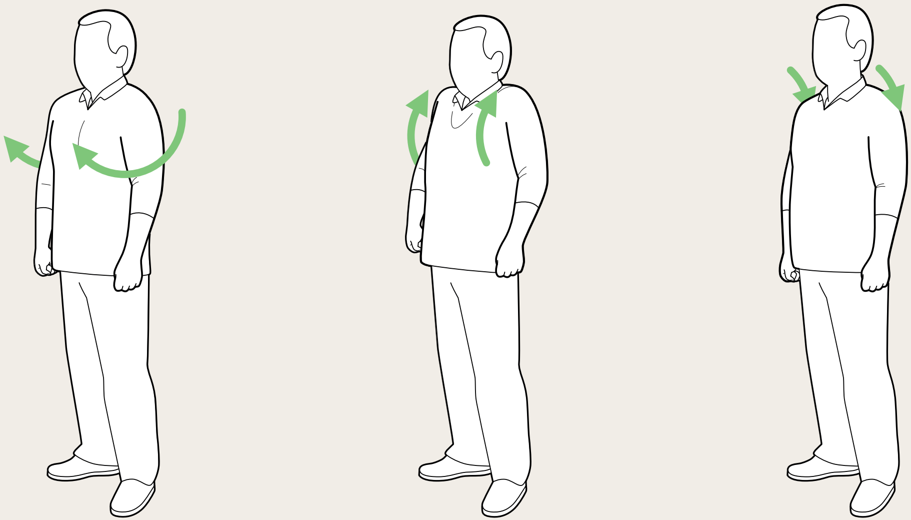

… then down and forward.

Lift them up and roll them back to your starting position. Relax for a few seconds then roll your shoulders back in the other direction.

Session
A

Cooling down
Exercise 5 - Weight on front foot

Put one foot forward while putting both hands against the wall. Keep your heels on the floor.

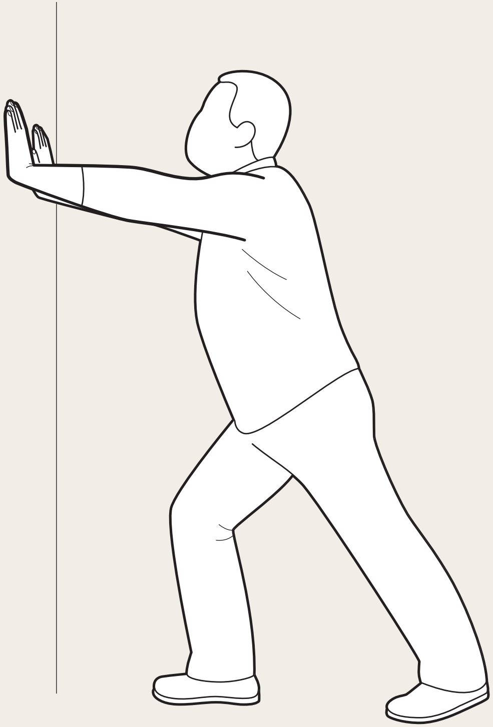

3×3
Repetitions

Do 3 sets of 3 repetitions. Rest for 2 minutes after each set.
If you find the exercise too easy, you can do extra repetitions.

Well done! You have now completed session A.

Bend your knee that is closest to the wall until it is over your foot. Hold it for 8 seconds and then stand up straight. Repeat this with your other foot.

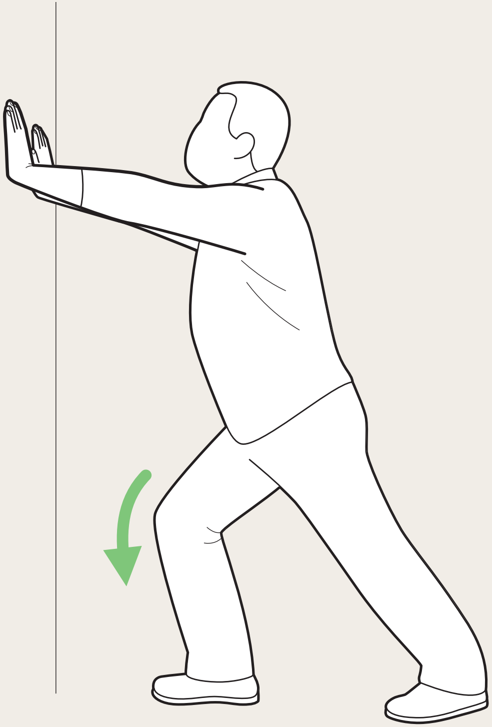

Session
B

Upper extremities

Exercise 1 - Front arm raise

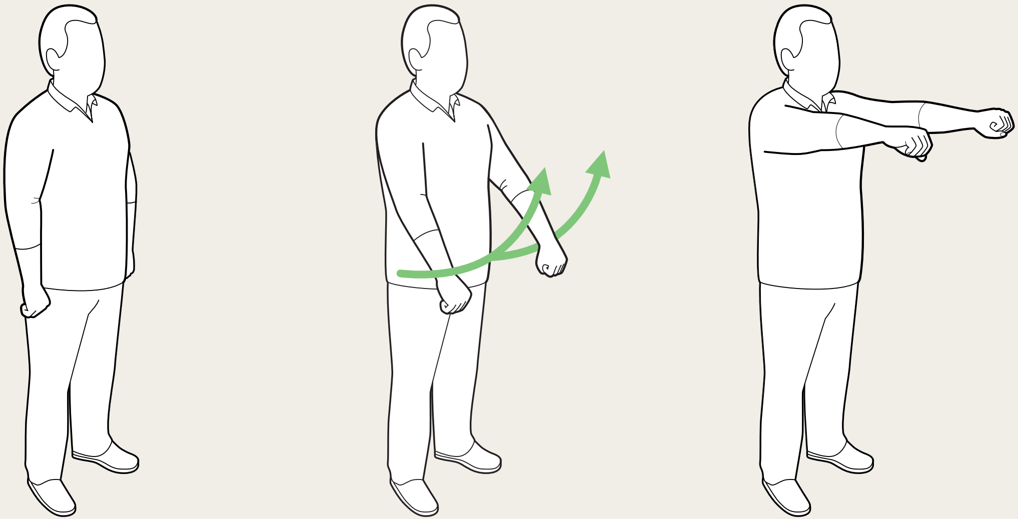

Stand up straight with your arms down at your sides with your palms facing backward.

While keeping them straight raise both of your arms up to shoulder height.

3×8
Repetitions

Do 3 sets of 8 repetitions. Rest for 1 minute after each set.
If you find the exercise too easy, you can do extra repetitions.

When you are finished, turn the page for your next exercise.

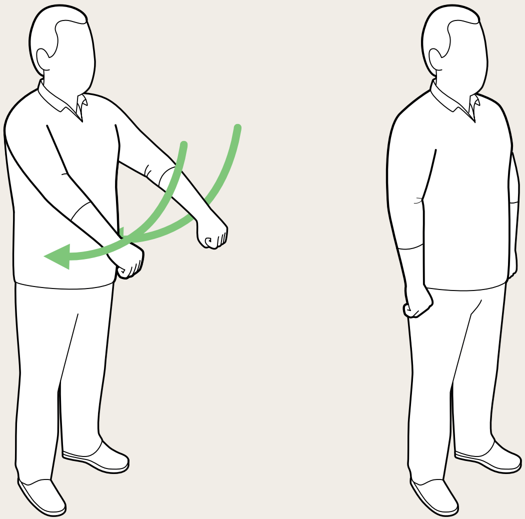

Once they reach your shoulder lower your arms back to your sides while keeping them straight. Bring them back to starting position.

Session
B

Lower extremities

Exercise 2 - Step up

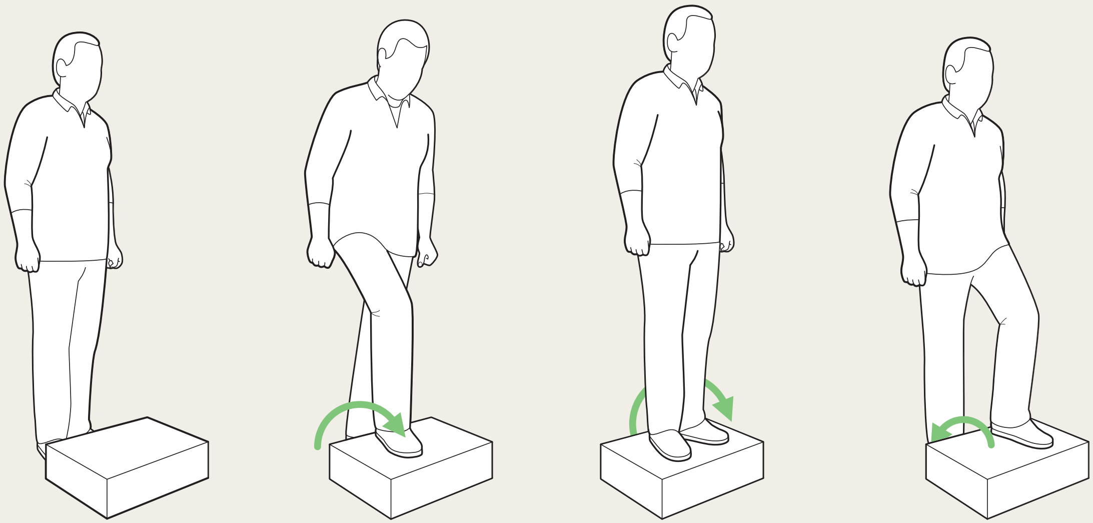

Stand up straight in front of a block or at the bottom of a set of stairs.

With your right foot take a step and immediately follow with your left foot.

Step down backwards starting with your right foot…

3×8

Repetitions

Do 3 sets of 8 repetitions. Rest for 2 minutes after each set.
If you find the exercise too easy, you can do extra repetitions.

When you are finished, turn the page for your next exercise.

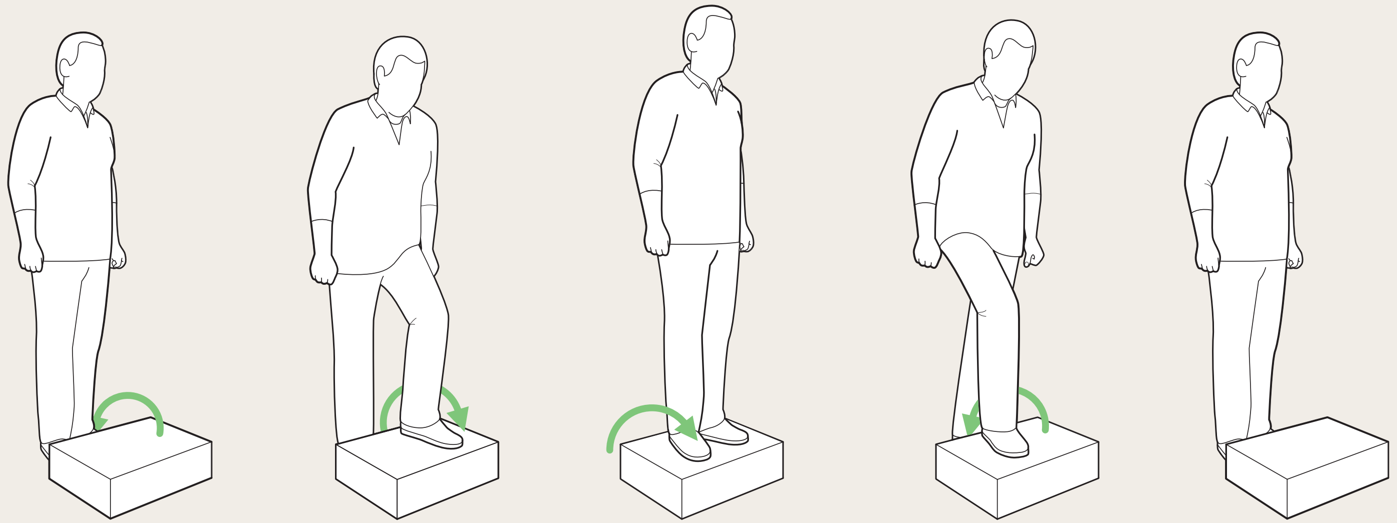

…and then immediately with your left foot.

Step up with your left foot and then your right foot.

Step down backwards starting with your left foot. Finish by standing up straight.

Session
B

Walking exercises
Exercise 3 - Side step

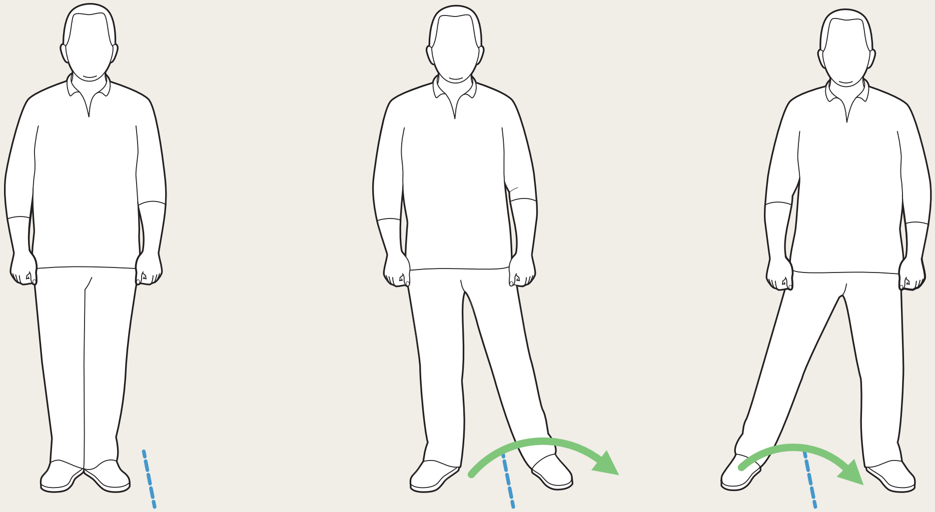

Stand up straight with both feet next to each other. Imagine there is a straight line next to your left foot.

While keeping your body in the same position step over the imaginary line with your left foot followed by your right foot.

3×8

Repetitions

Do 3 sets of 8 repetitions. Rest for 1 minute after each set.
If you find the exercise too easy, you can do extra repetitions.

When you are finished, turn the page for your next exercise.

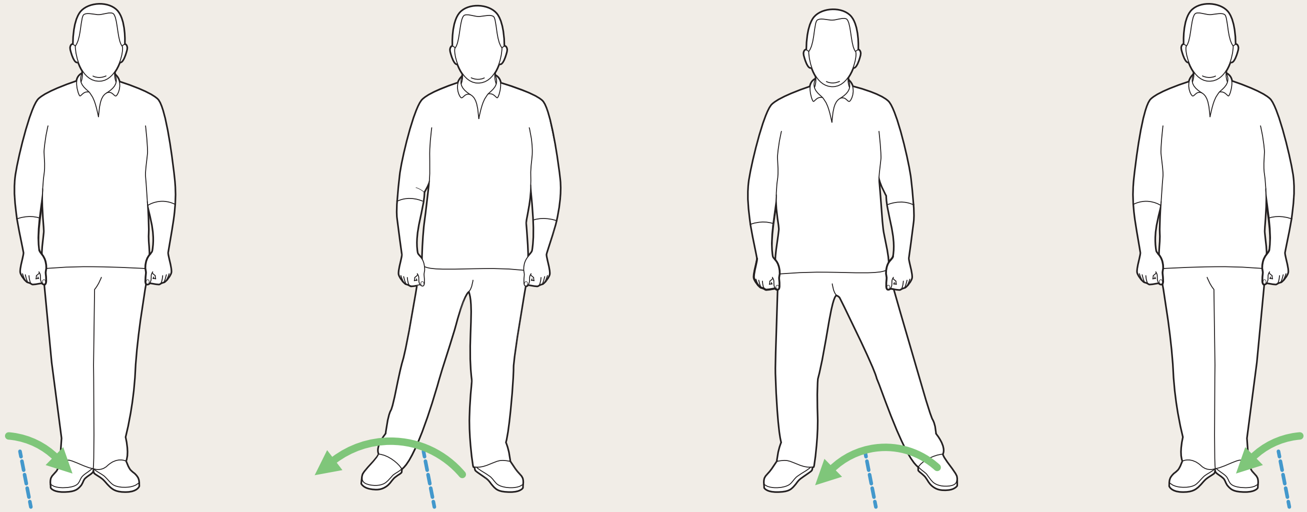

Both feet are now next to one another and the imaginary line is next to your right foot.

Step over the imaginary line with your right foot while looking straight ahead and follow with your left foot.

Finish by standing up straight.

Session

B

Cooling down

Exercise 4 - Shoulder shrug

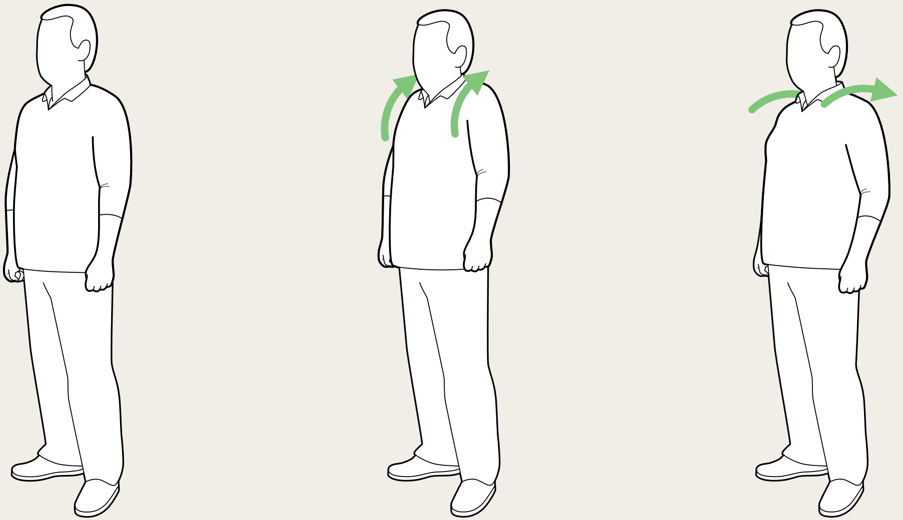

Start by standing with your arms by your side.

Slowly lift your shoulders up and roll them back...

3×5
Repetitions

Do 3 sets of 5 repetitions. Rest for 2 minutes after each set.
If you find the exercise too easy, you can do extra repetitions.

When you are finished, turn the page for your next exercise.

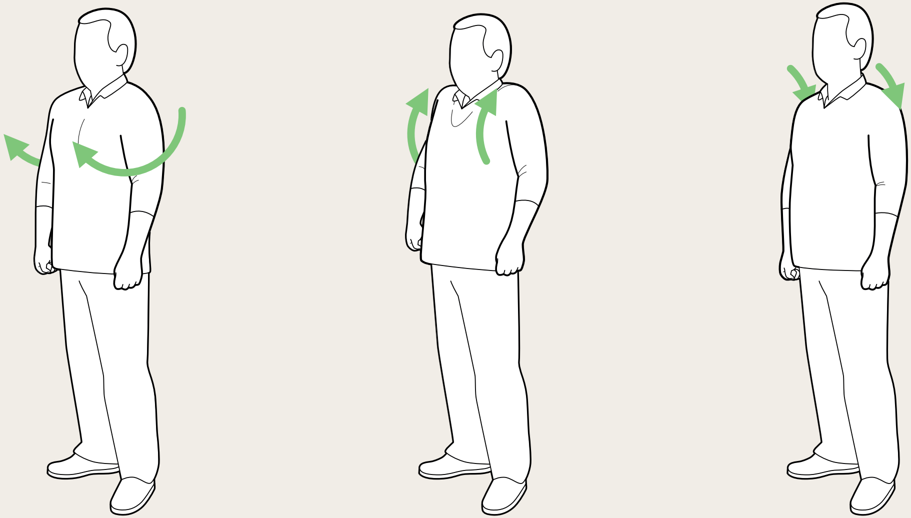

… then down and forward.

Lift them up and roll them back to your starting position. Relax for a few seconds then roll your shoulders back in the other direction.

Session

B

Cooling down

Exercise 5 - Foot grab

Stand behind a chair. Hold onto the chair with your left hand. While bending your right knee grab your right foot with your other hand. Pull your leg until you feel a stretch in your thigh and hold it for 8 seconds.

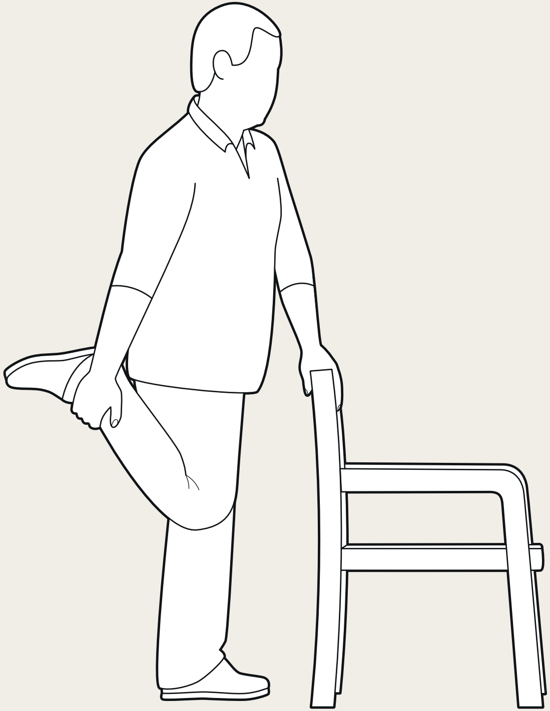

3×3
Repetitions

Do 3 sets of 3 repetitions. Rest for 2 minutes after each set.
If you find the exercise too easy, you can do extra repetitions.

Well done! You have now completed session B.

Put your right foot down and hold on to the chair with right hand. Bend your left knee and grab your foot with your left hand. Gently pull your leg until you feel a stretch in your thigh and hold it for 8 seconds.

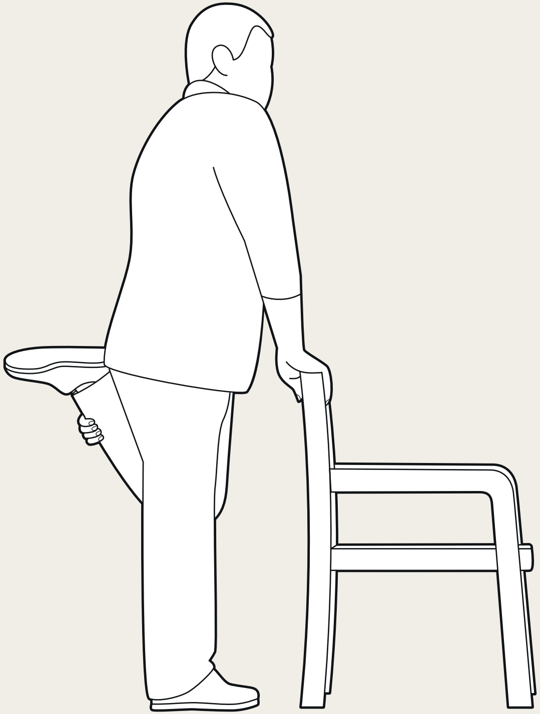

Session

C

Upper extremities

Exercise 1 - Side arm raise

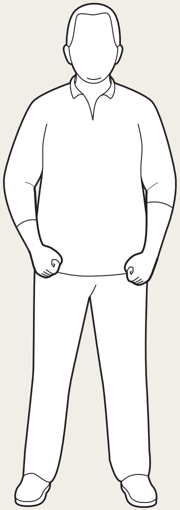

Stand up straight while holding your arms down at your side with your palms facing inward.

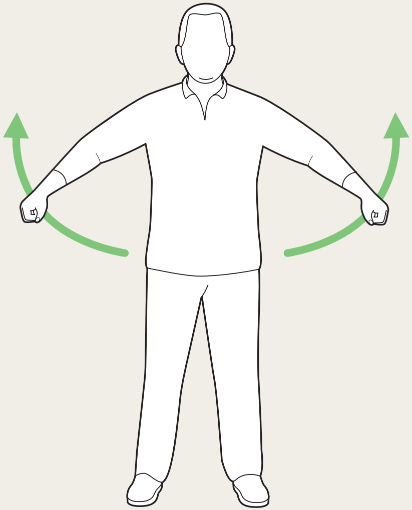

Raise both arms up to shoulder height.

3×8
Repetitions

Do 3 sets of 8 repetitions. Rest for 1 minute after each set.
If you find the exercise too easy, you can do extra repetitions.

When you are finished, turn the page for your next exercise.

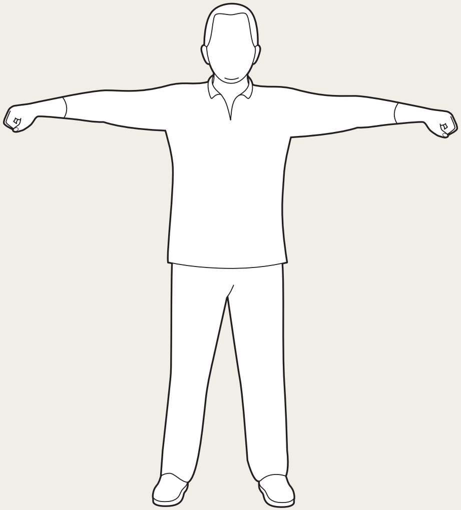

Hold this position for 1 second.

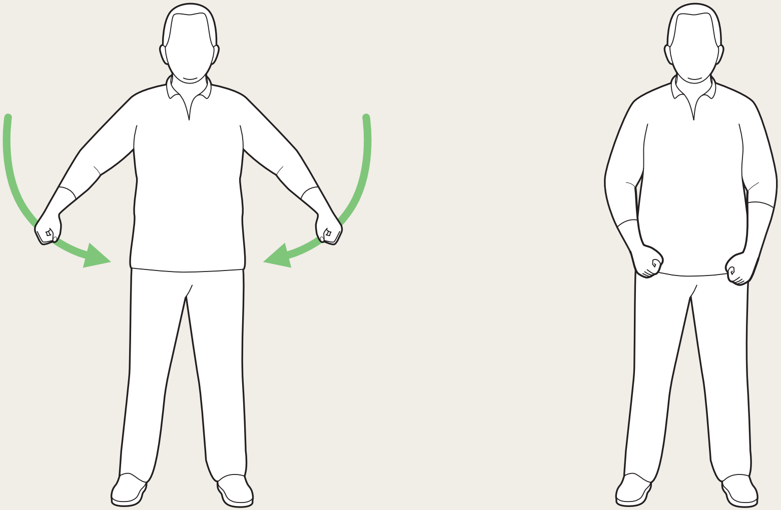

Lower your arms and return to the starting position.

Session

C

Lower extremities

Exercise 2 - Knee curl

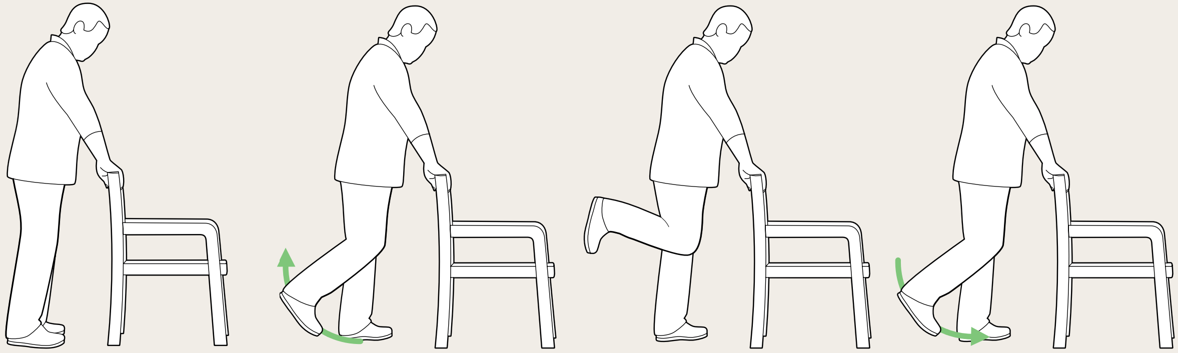

Stand behind a chair. Hold the back of the chair for balance.

Lift your right foot by bending your knee. Keep your hips still.

Lift the heel of your right foot as high as possible.

Lower your right foot back down to the floor.

3×8

Repetitions

Do 3 sets of 8 repetitions. Rest for 1 minute after each set.
If you find the exercise too easy, you can do extra repetitions.

When you are finished, turn the page for your next exercise.

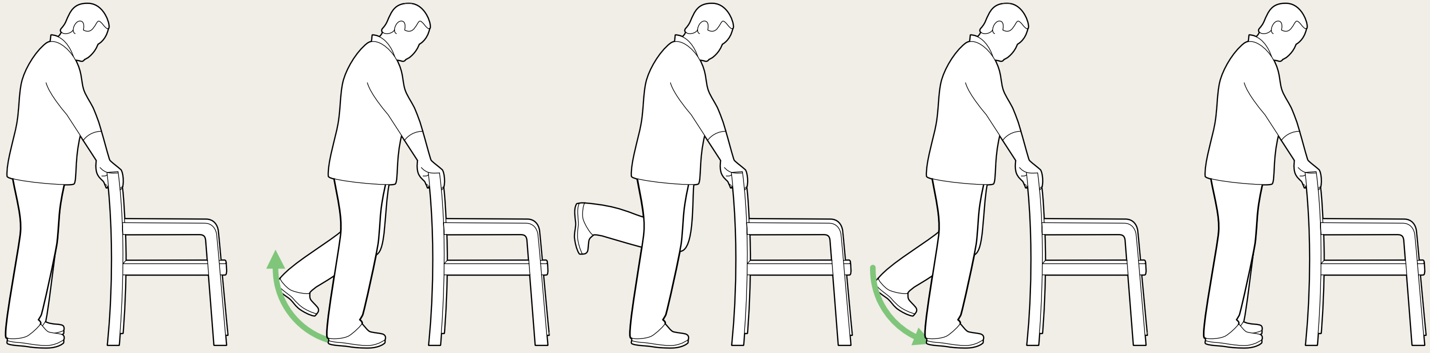

Lift your left foot by bending your knee. Remember to keep your hips still.

Lift the heel of your left foot as high as possible.

Lower your left foot back down to the floor and stand up straight.

Session
C

Walking exercises

Exercise 3 - High knee walking

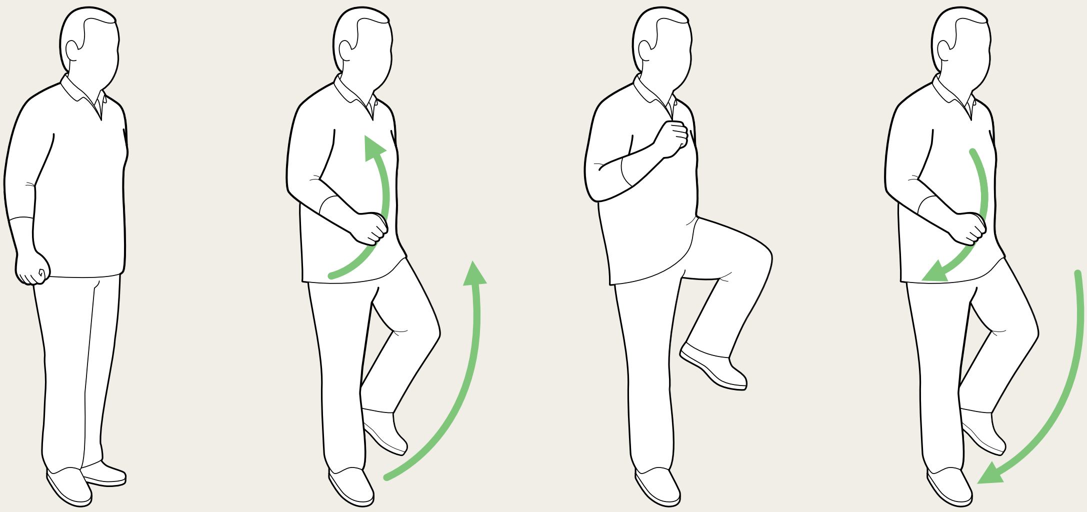

Stand up straight with your feet next to each other.

Lift up your left knee and raise your right hand by bending your right elbow.

Lift up your knee as high as possible while moving your right hand towards your right shoulder.

Put down your knee and hand.

2×1
Minutes

Do 2 sets of 1 minute. Rest for 1 minute after each set.
If you find the exercise too easy, you can do it for longer.

When you are finished, turn the page for your next exercise.

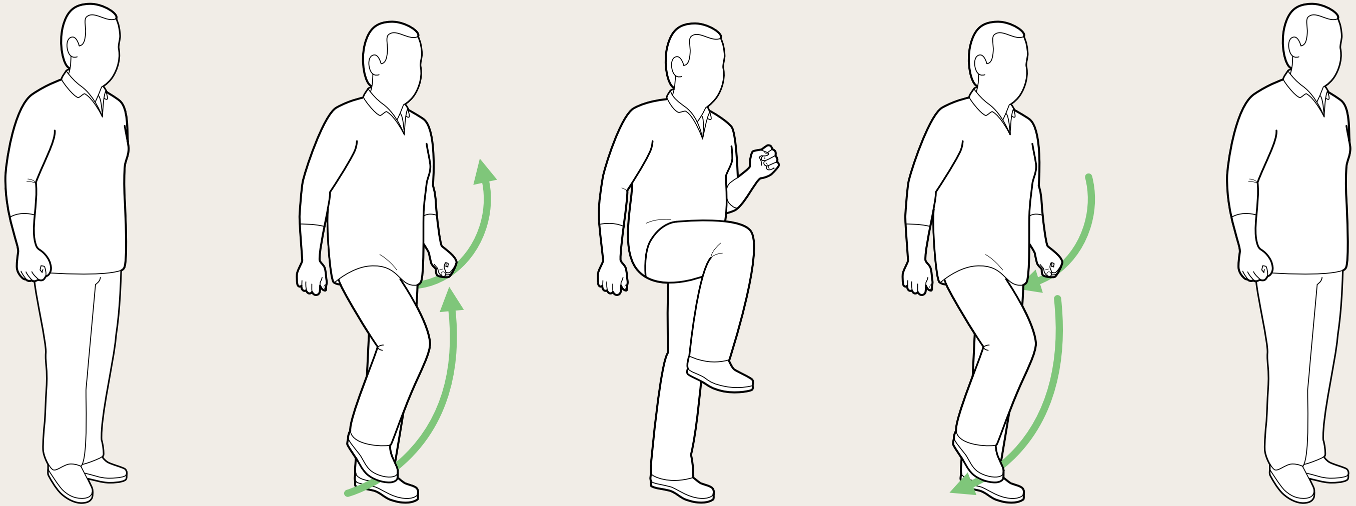

Lift up your right knee and raise your left hand by bending your left elbow.

Lift up your knee as high as possible while moving your hand towards your left shoulder.

Lower your knee and your hand.

Session

C

Cooling down

Exercise 4 - Weight on front foot

Put one foot forward while putting both hands against the wall and keep your heels on the floor.

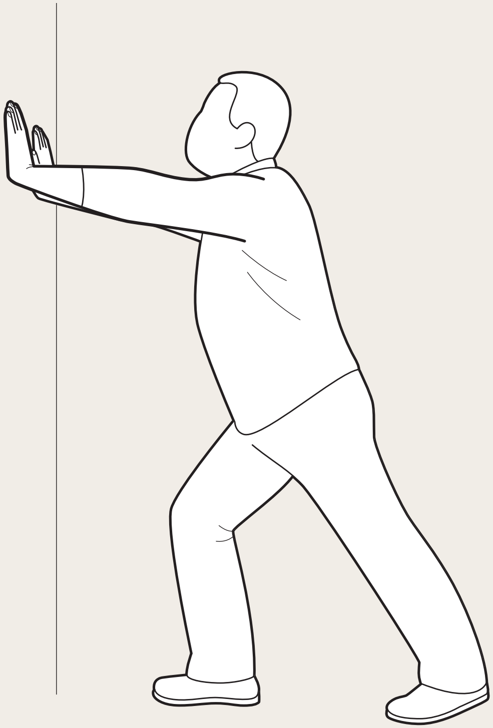

3×3 Repetitions

Do 3 sets of 3 repetitions. Rest for 2 minutes after each set.
If you find the exercise too easy, you can do extra repetitions.

When you are finished, turn the page for your next exercise.

Bend your knee that is closest to the wall until it is over your foot. Hold it for 8 seconds and then stand up straight. Reposition and repeat these steps with your other foot.

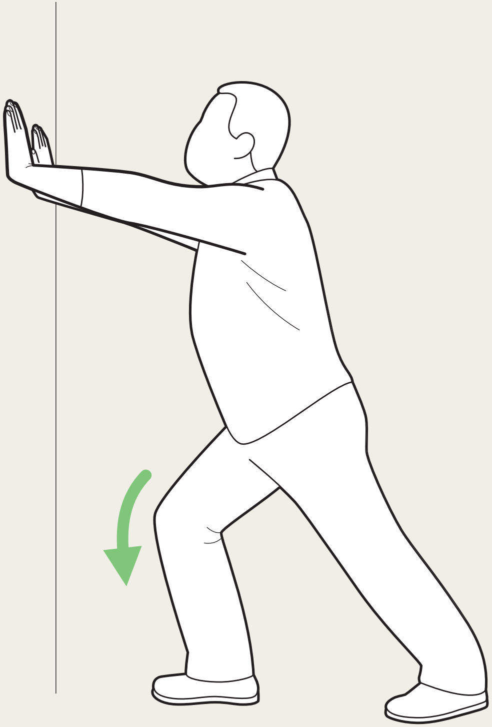

Session C

Cooling down

Exercise 5 - Leg stretch

While sitting on a chair stretch your left leg straight out keeping your heel on the floor. Bend your right leg and keep your right foot flat on the floor. Bend forward from your hip and try and touch your toes. Hold this position for 8 seconds.

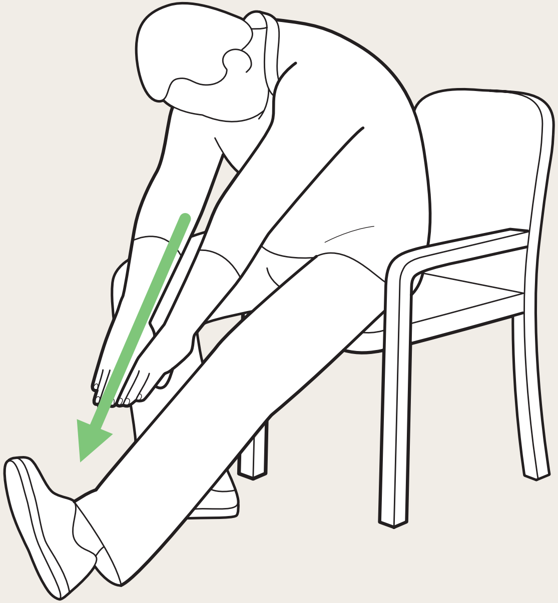

3×3
Repetitions

Do 3 sets of 3 repetitions. Rest for 2 minutes after each set.
If you find the exercise too easy, you can do extra repetitions.

Well done! You have now completed session C.

Start with your other leg by stretching your right leg out in front of you with your heel on the floor. Bend your left leg so that your foot is flat on the floor and slowly bend forward as far as you can. Hold this position for 8 seconds.

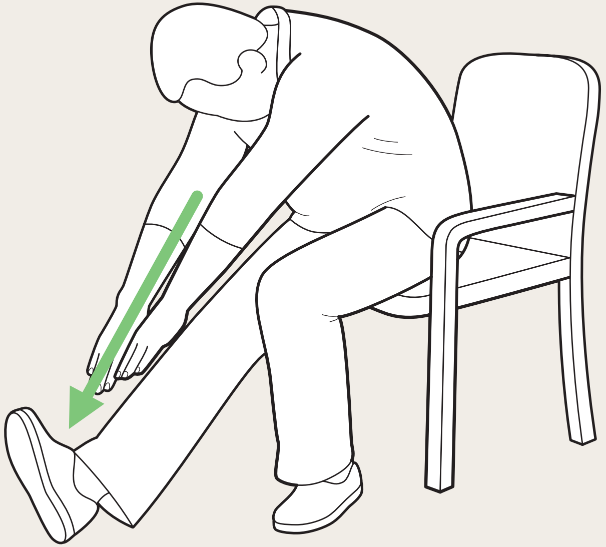

SPACE FOR IMPRINT/IMPRESSUM
