# Supplementary material for: Smartphone-Based Physical Activity Telecoaching in Chronic Obstructive Pulmonary Disease: Mixed-Methods Study on Patient Experiences and Lessons for Implementation
Source: JMIR Mhealth Uhealth. 2018 Dec 21;6(12):e200. doi: 10.2196/mhealth.9774 (PMC6320438; doi:10.2196/mhealth.9774)
Supplement: Multimedia Appendix 2 [file mhealth_v6i12e200_app2.pptx]

## Slide 1
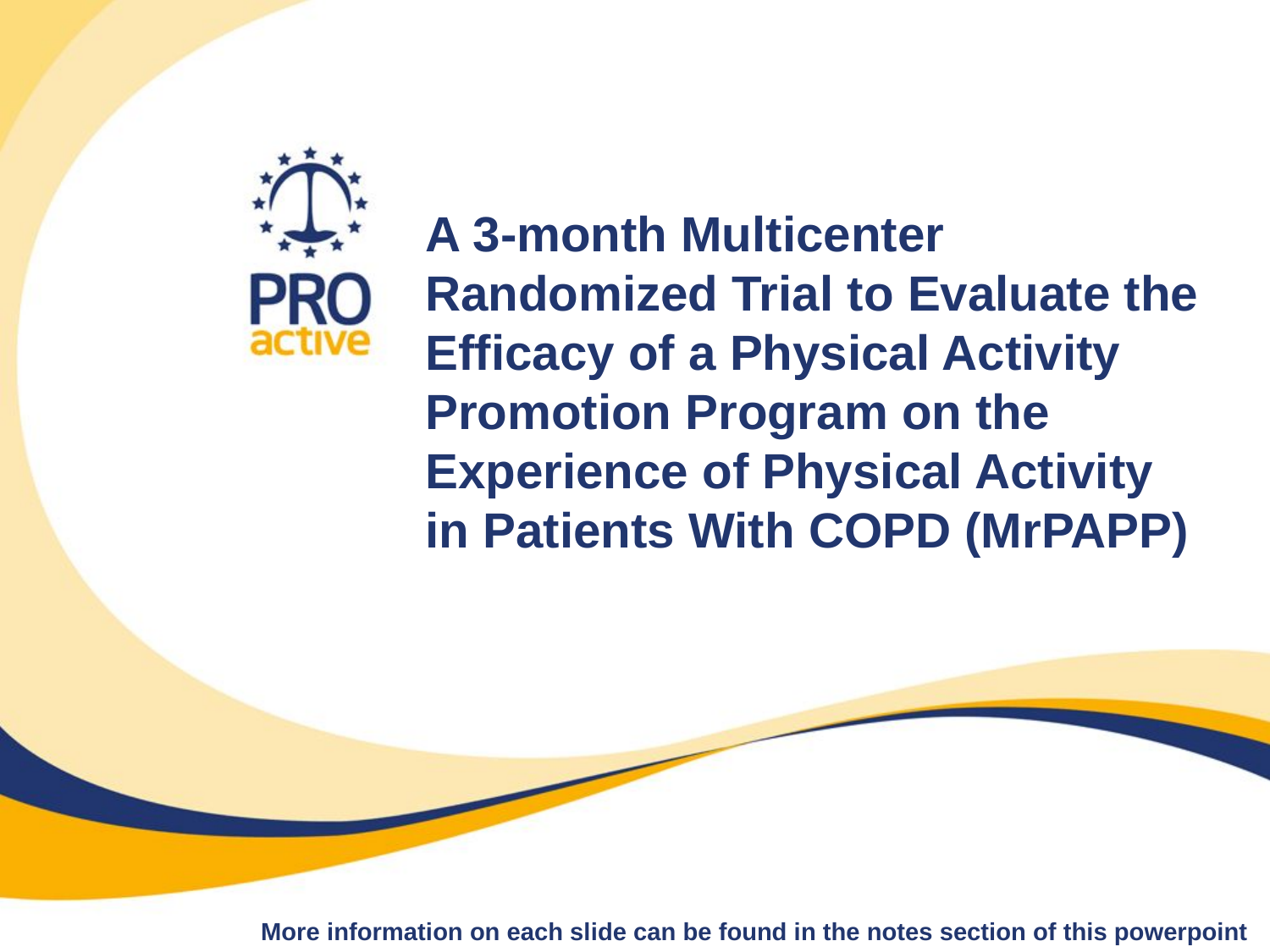

A 3-month Multicenter Randomized Trial to Evaluate the Efficacy of a Physical Activity Promotion Program on the Experience of Physical Activity in Patients With COPD (MrPAPP)
More information on each slide can be found in the notes section of this powerpoint

## Slide 2
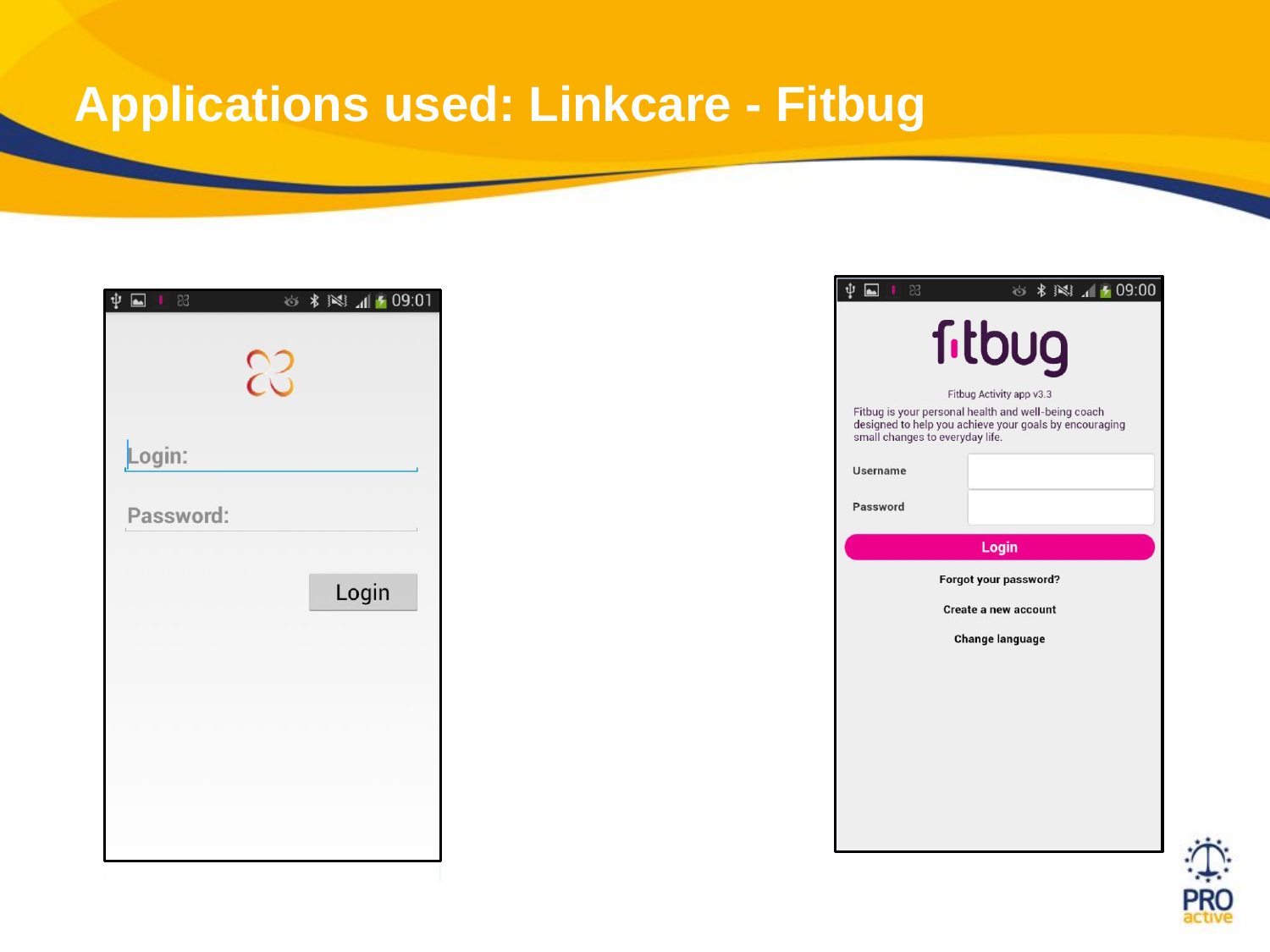

# Applications used: Linkcare - Fitbug

## Slide 3
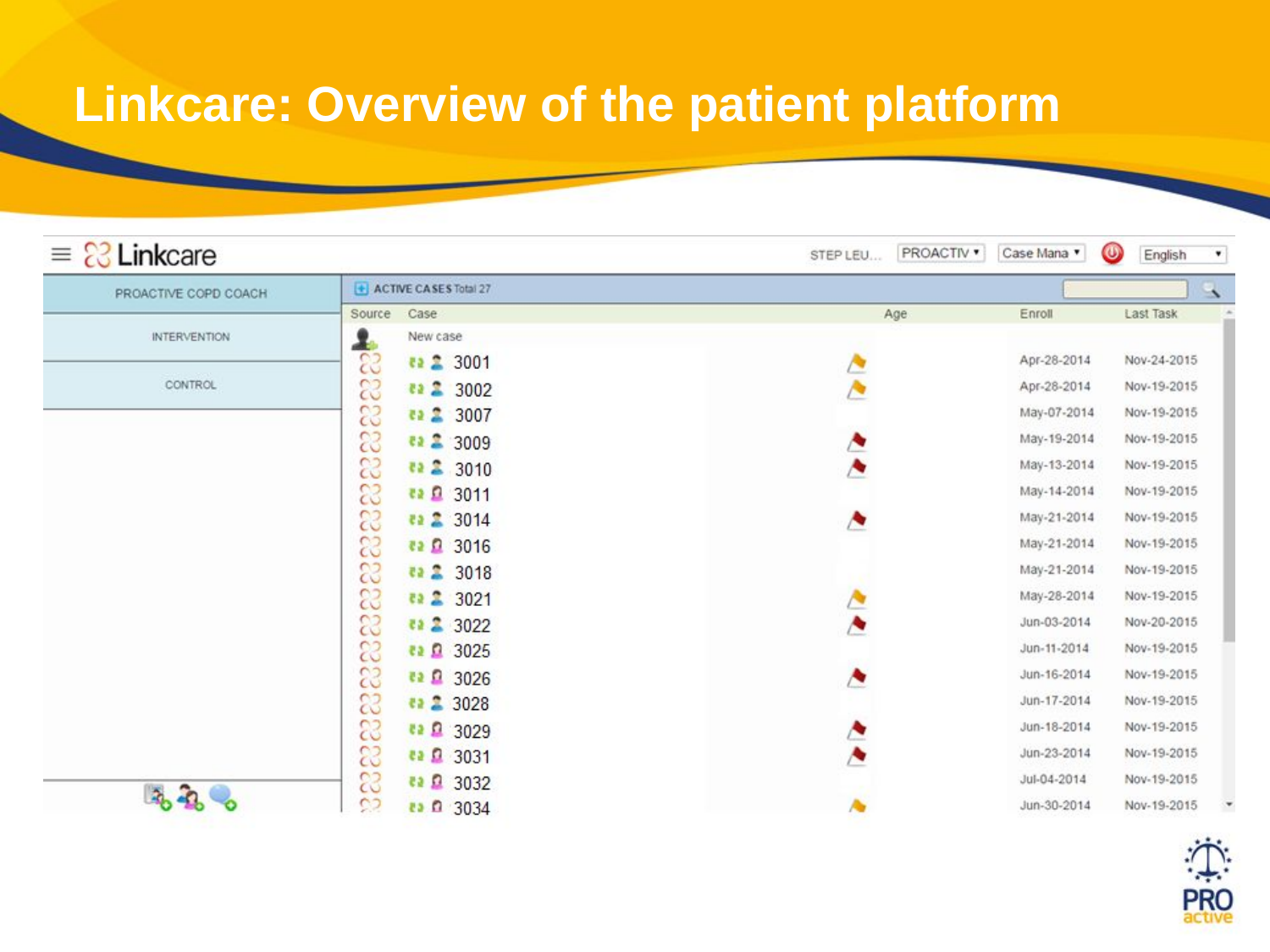

# Linkcare: Overview of the patient platform

## Slide 4
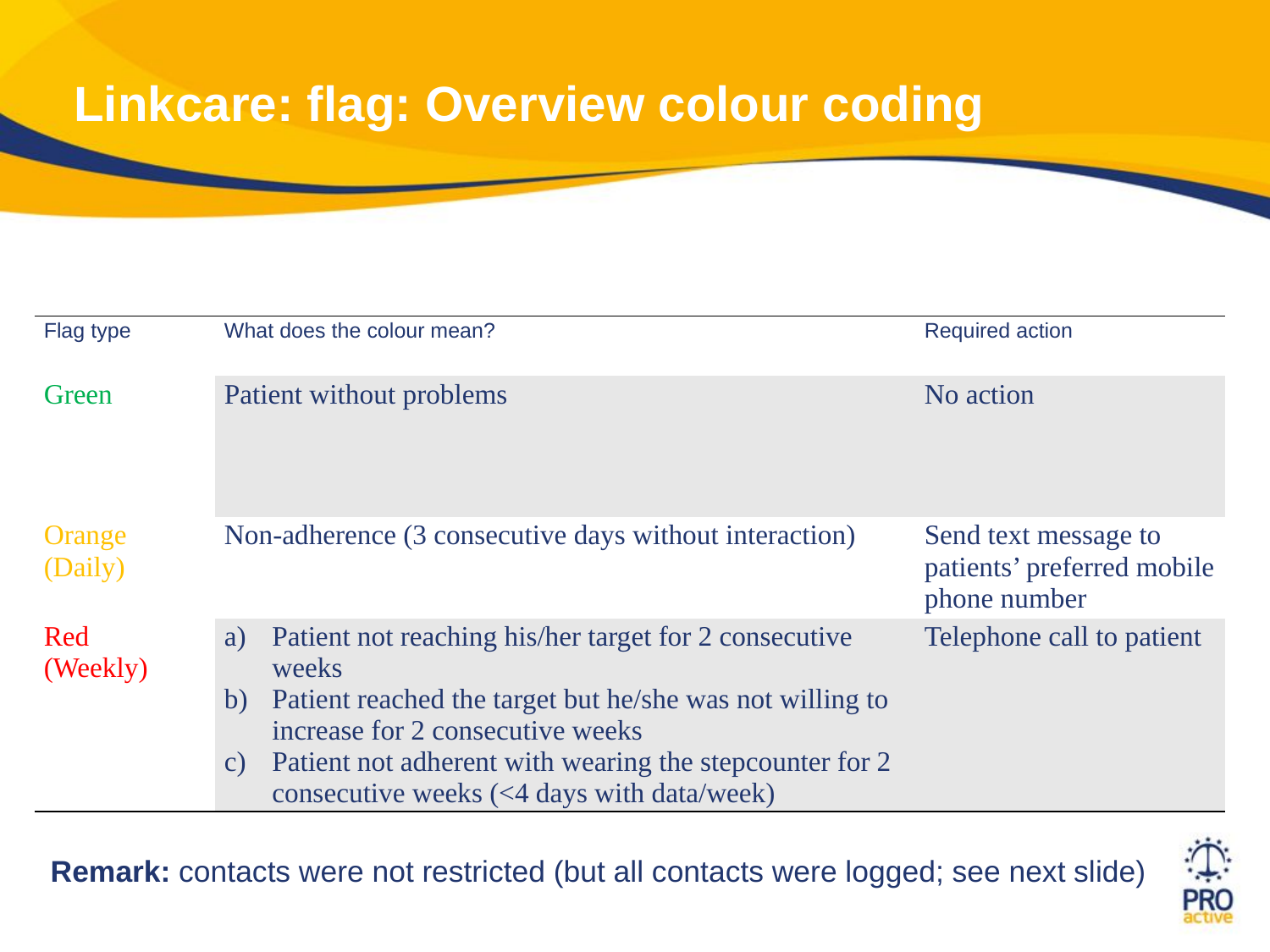

# Linkcare: flag: Overview colour coding
| Flag type | What does the colour mean? | Required action |
| --- | --- | --- |
| Green | Patient without problems | No action |
| Orange (Daily) | Non-adherence (3 consecutive days without interaction) | Send text message to patients’ preferred mobile phone number |
| Red (Weekly) | Patient not reaching his/her target for 2 consecutive weeks Patient reached the target but he/she was not willing to increase for 2 consecutive weeks Patient not adherent with wearing the stepcounter for 2 consecutive weeks (<4 days with data/week) | Telephone call to patient |
Remark: contacts were not restricted (but all contacts were logged; see next slide)

## Slide 5
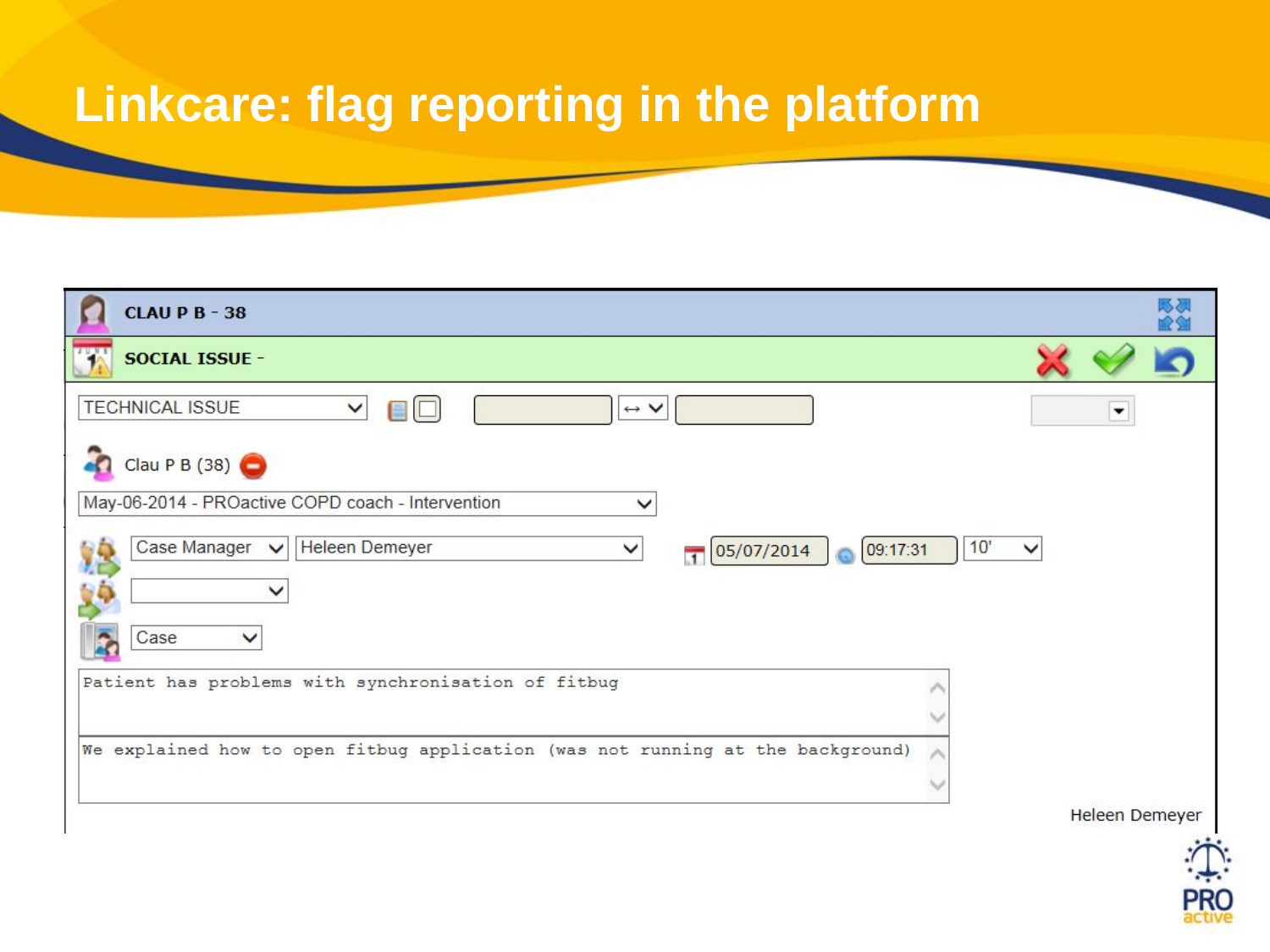

# Linkcare: flag reporting in the platform

## Slide 6
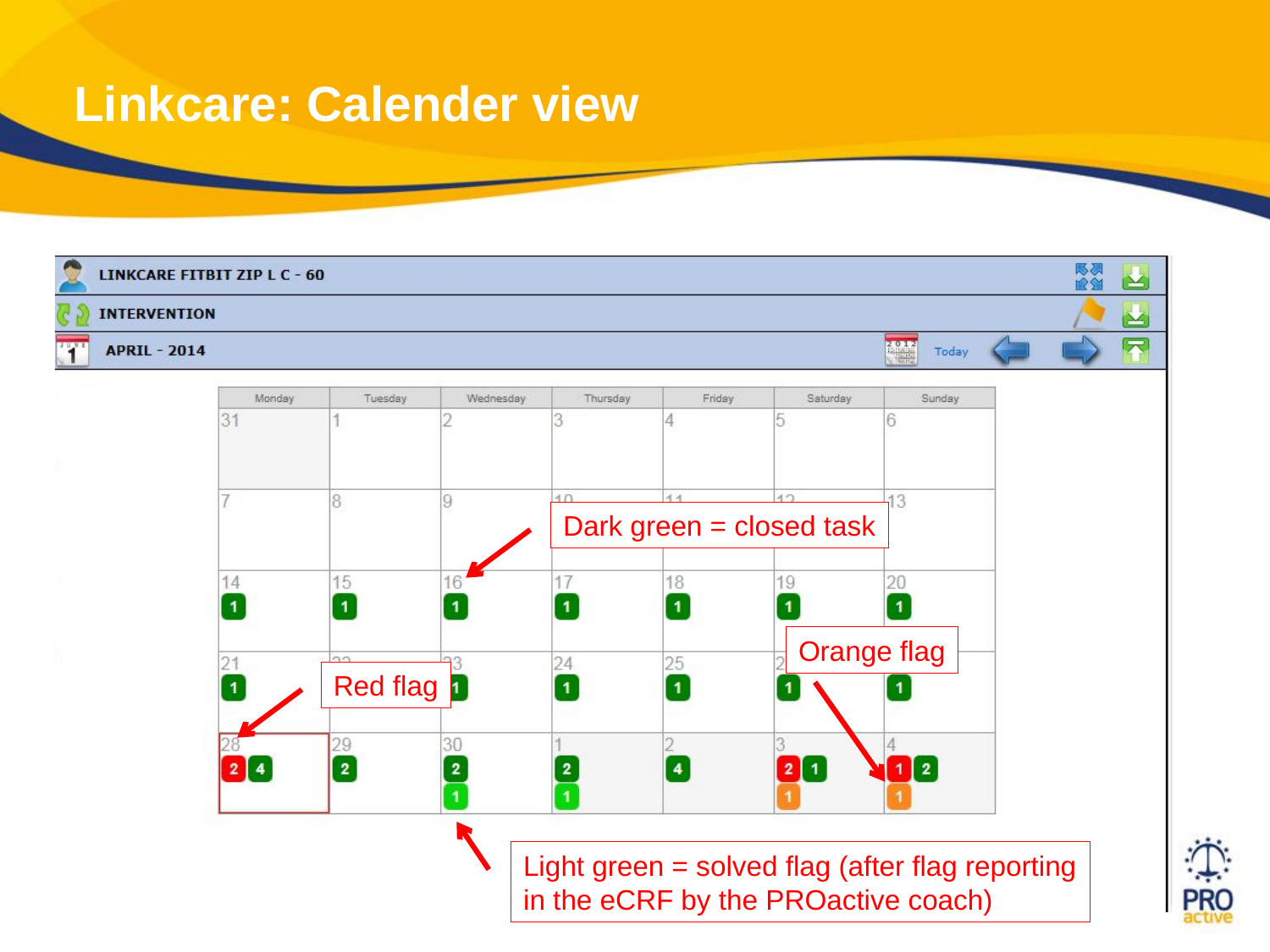

# Linkcare: Calender view
Dark green = closed task
Orange flag
Red flag
Light green = solved flag (after flag reporting
in the eCRF by the PROactive coach)

## Slide 7
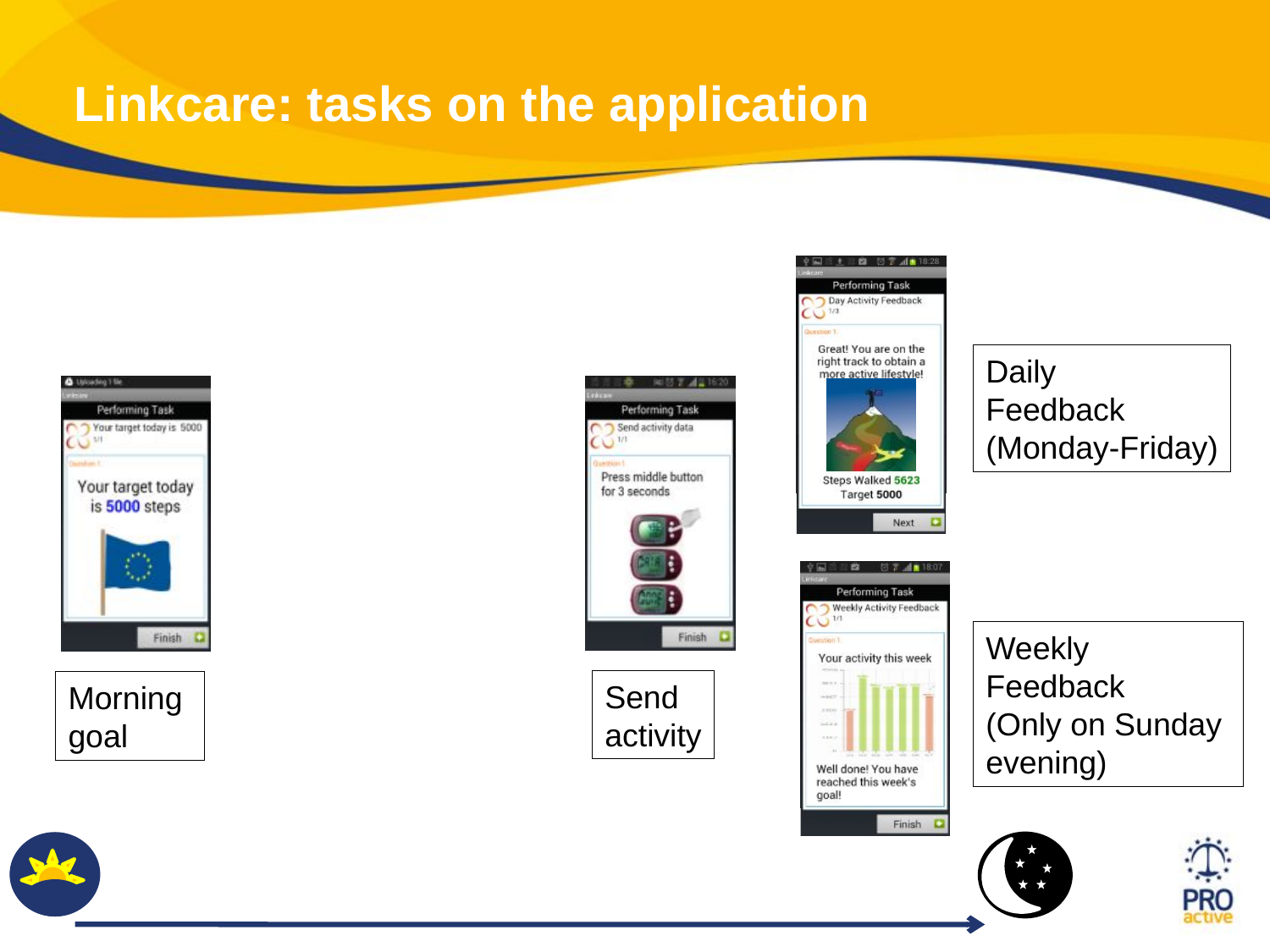

# Linkcare: tasks on the application
Daily
Feedback
(Monday-Friday)
Weekly
Feedback
(Only on Sunday
evening)
Send
activity
Morning
goal

## Slide 8
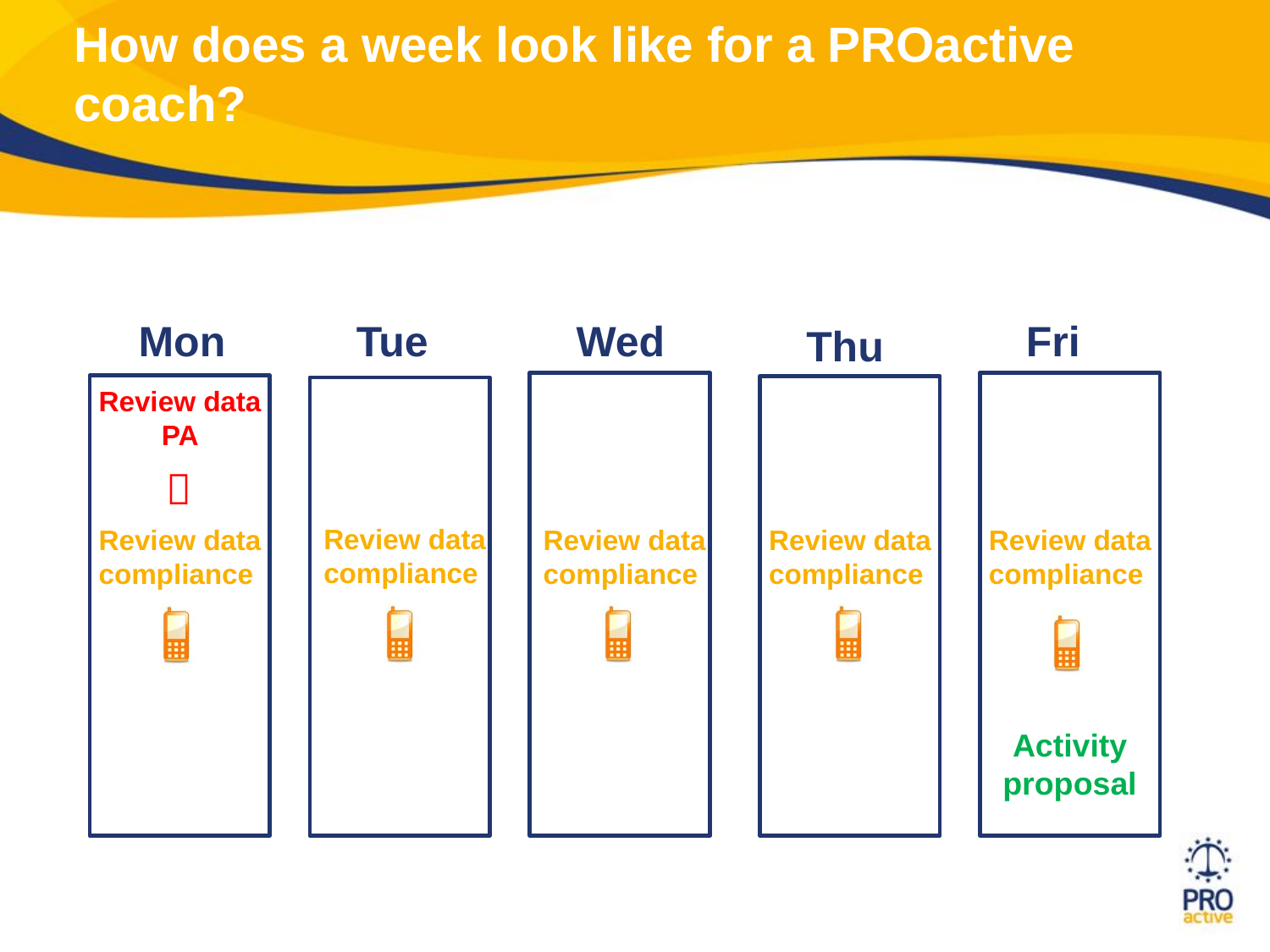

# How does a week look like for a PROactive coach?
Fri
Mon
Wed
Tue
Thu
Review data
PA

Review data
compliance
Review data
compliance
Review data
compliance
Review data
compliance
Review data
compliance
Activity proposal

## Slide 9
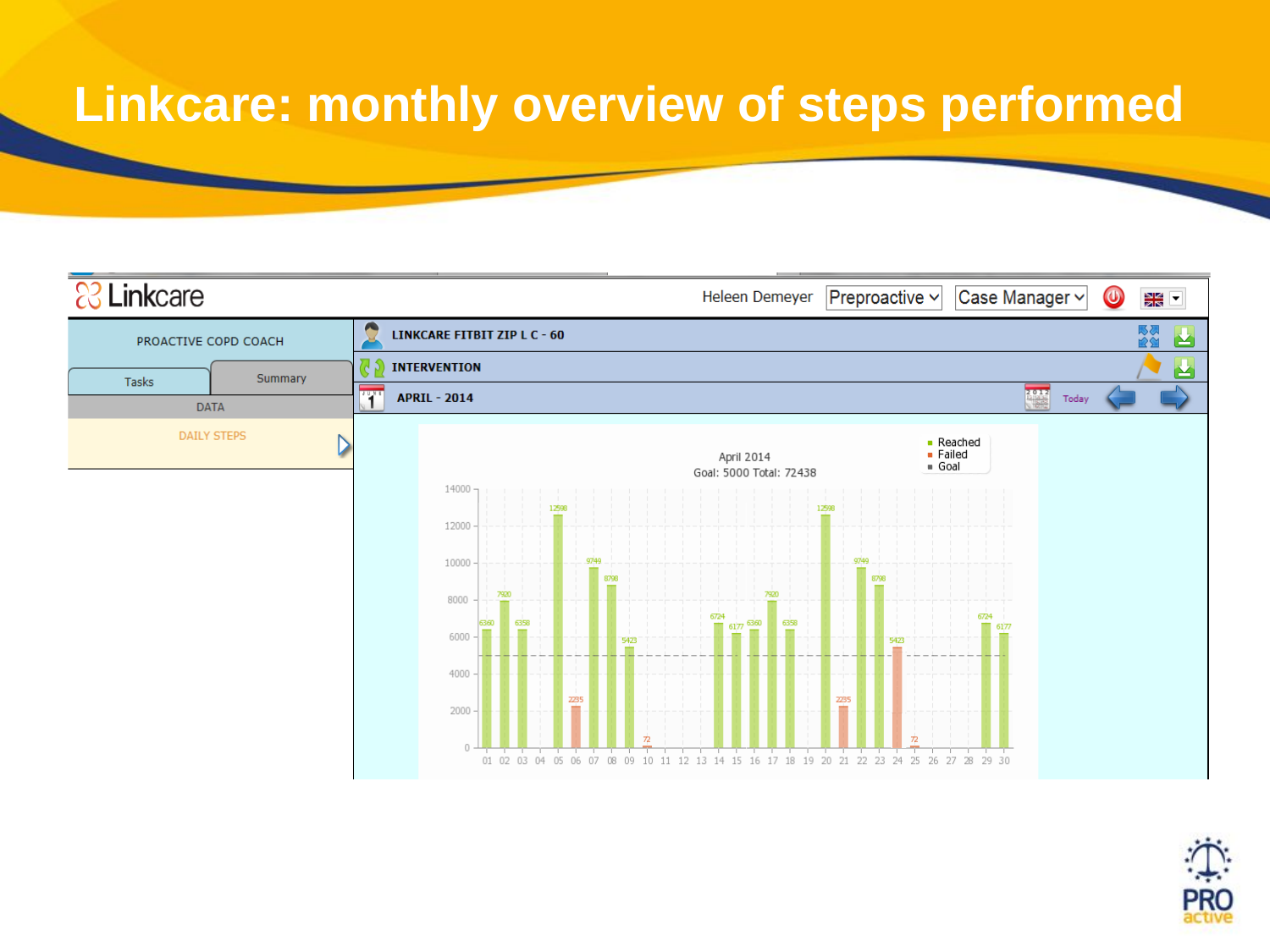

# Linkcare: monthly overview of steps performed
