# Supplementary material for: Smartphone-Based Physical Activity Telecoaching in Chronic Obstructive Pulmonary Disease: Mixed-Methods Study on Patient Experiences and Lessons for Implementation
Source: JMIR Mhealth Uhealth. 2018 Dec 21;6(12):e200. doi: 10.2196/mhealth.9774 (PMC6320438; doi:10.2196/mhealth.9774)
Supplement: Multimedia Appendix 3 [file mhealth_v6i12e200_app3.pdf]

Patientid:
Date:

WP6 Mr PaPP study

Patient satisfaction form

Dear,

During the last 3 months you have participated in the coaching program of the PROactive study. This coaching program included a telecoaching system developed for this specific intervention. By taking part in this study you are now well placed to evaluate this coaching program. Your experiences during the intervention can help us to further improve this coaching program in the future. Therefore we would like to have your opinion about the intervention and we kindly ask you to take some time to complete this patient satisfaction form. Your input is highly appreciated! Many thanks again for participating in this study and sharing your experiences with us.

All the best,

The PROactive team

Patientid:
Date:

How much did you **enjoy taking part** in this activity program?

- I liked it a lot
- I liked it
- Neutral
- I did not like it
- No opinion

Did the intervention **coach you in increasing** your physical activity?

- Yes, it helped me a lot
- Yes, a little bit
- Not noticeable
- No, not at all
- No, it rather discouraged me

How did you **experience the weekly increases** proposed during the intervention?

- Much too low
- A little bit too low
- Reasonable
- A little bit too high
- Much too high

How was it for you to work with the **smartphone intervention**?

- Very easy
- Easy
- Not easy, but I managed
- Difficult
- Very difficult

What was for you the **most important part** of the intervention?

- The step counter
- The application on the smartphone
- The text messages
- The home exercise booklet
- The activity leaflet (general information about physical activity)
- The telephone contacts with the study team
- Other (please specify)……………………………………………………………………………………………………

Patientid:
Date:

How useful did you find the following parts of the intervention for increasing your PA?

1) The **step counter**

0 1 2 3 4 5 6 7 8 9 10
Not useful at all — Very useful

2) Daily **activity goal** displayed on your smartphone during the day

0 1 2 3 4 5 6 7 8 9 10
Not useful at all — Very useful

- I did not receive activity goals in the morning

3) Activity **feedback** in the evening (display of text about your achievement together with a picture)

0 1 2 3 4 5 6 7 8 9 10
Not useful at all — Very useful

- I did not receive activity feedback in the evening

4) **Graph displaying** your achievements of the week

0 1 2 3 4 5 6 7 8 9 10
Not useful at all — Very useful

- I did not receive a graph displaying my achievements

5) **Tip of the day**

0 1 2 3 4 5 6 7 8 9 10
Not useful at all — Very useful

- I did not receive any tips about physical activity

6) **Text messages** sent by your study team

0 1 2 3 4 5 6 7 8 9 10
Not useful at all — Very useful

- I did not receive text messages of my study team

7) **Phone calls** with the study team

0 1 2 3 4 5 6 7 8 9 10
Not useful at all — Very useful

- I did not have any telephone contact with the study team

8) **Home exercise** booklet

0 1 2 3 4 5 6 7 8 9 10
Not useful at all — Very useful

- I did not receive a booklet with home exercises

Patientid:
Date:

How often did you (in general) perform the following actions?

	Several times per day	Once per day	Sometimes, but not every day	Once or twice per week	Never
Look at your stepcounter **during the day**					
Do the **home exercises**					

How would you rate the graphics used in the Linkcare-application

- Very helpful and intuitive/supportive
- Helpful and intuitive/supportive
- Neutral
- Poor, not intuitive/supportive
- Very poor, not intuitive/supportive at all

Comment:

……….

……….

……….

How would you rate the interaction between you and the app

- Very quick
- Quick
- Neutral
- Slow
- Very slow

Comment:

……….

……….

……….

Patientid:
Date:

Which part of the intervention would you be willing to use further in the future?

- Nothing
- The stepcounter
- The stepcounter and the mobile phone providing feedback messages
- The stepcounter, the mobile phone and the contact with the study team
- The whole intervention

I used the application by means of

- Telephone network
- My wireless at home
- Both wireless and the telephone network

Would you like to add a comment?

…….

…….

…….

…….

…….

Thank you for your time and we wish you all the best!
