# Supplementary material for: Smartphone-Based Physical Activity Telecoaching in Chronic Obstructive Pulmonary Disease: Mixed-Methods Study on Patient Experiences and Lessons for Implementation
Source: JMIR Mhealth Uhealth. 2018 Dec 21;6(12):e200. doi: 10.2196/mhealth.9774 (PMC6320438; doi:10.2196/mhealth.9774)
Supplement: Multimedia Appendix 4 [file mhealth_v6i12e200_app4.pdf]

Patientid:
Date:

WP6 Mr PaPP study

Discussion guide

1) How did you **experience** the intervention?
……
……
……
……
2) How did you experience the **technical aspects** of the intervention? What were the difficulties you experienced? What was easy? (discuss the mobile phone, user-friendliness),
……
……
……
……
3) What did help/**support** you the most in coaching to a higher PA level?
……
……
……
4) Classify patients based on their achievement during the study (indicate classification)
   a) Success

   Aim is to discuss with patient motivation, experience, ways of achieving the goal.

   E.g. We can see an increase in your PA level. How does it looks for you? How did you do this? What was your motivation?

   b) No big change

   Aim is to discuss difficulties, *experience*
   E.g. We do not see a considerable change compared to the start of the study. How do you feel this?

   c) 'Relapse'
   Aim is to discuss the triggers for changes in PA level during the study by focusing of the 'relapse' periods.

……
……
……
……

Patientid:

Date:

………

………

………

………
