# Supplementary material for: Smartphone-Based Physical Activity Telecoaching in Chronic Obstructive Pulmonary Disease: Mixed-Methods Study on Patient Experiences and Lessons for Implementation
Source: JMIR Mhealth Uhealth. 2018 Dec 21;6(12):e200. doi: 10.2196/mhealth.9774 (PMC6320438; doi:10.2196/mhealth.9774)
Supplement: Multimedia Appendix 5 [file mhealth_v6i12e200_app5.pdf]

Date:

Focus group coaches

Part 1: investigator

a) Questions about appreciation

- *Was it feasible to perform every part of the intervention? Which parts did/didn't we conduct?*

- What is the general appreciation?
  - *What was good?*
  - *What could be improved?*

- What is the appreciation of
  - *Stepcounter*
  - *Application (Linkcare and fitbug)*
  - *Home exercise booklet*
  - *Text messages*
  - *Phone calls with patients*

- *What could be reasons of low actual usage in this patient population?*
- *Do you find this telecoaching intervention useful in addition to the usual therapy in patients with COPD?*
- *What would you be willing to continue using in the future and for how long?*
- *What would you change for future use?*
- *How long did it take to*
  - *Manage the interface? (Linkcare)*
  - *Solve technical problems of patients?*

Date:

- *How many patients would you be able to manage at this moment?*
