# Supplementary material for: Smartphone-Based Physical Activity Telecoaching in Chronic Obstructive Pulmonary Disease: Mixed-Methods Study on Patient Experiences and Lessons for Implementation
Source: JMIR Mhealth Uhealth. 2018 Dec 21;6(12):e200. doi: 10.2196/mhealth.9774 (PMC6320438; doi:10.2196/mhealth.9774)
Supplement: Multimedia Appendix 6 [file mhealth_v6i12e200_app6.pdf]

Consolidated criteria for reporting qualitative studies (COREQ): interview MrPapp trial (NCT02158065)

Developed from:
Tong A, Sainsbury P, Craig J. Consolidated criteria for reporting qualitative research (COREQ): a 32-item checklist. *International Journal for Quality in Health Care*. 2007. Volume 19, Number 6: pp. 349 – 357

**No. Item**	**Guide questions/description**	
**Domain 1: Research team and reflexivity**		
Personal Characteristics		
1. Inter viewer/facilitator	Which author/s conducted the interview?	Nine authors conducted the interviews:<br>ML (male; physiotherapist; Msc)<br>HD (female; physiotherapist, PhD)<br>ZL (male; exercise physiologist, PhD)<br>RT (female, physiotherapist; Msc)<br>NR (male; physiotherapist; Msc)<br>AF (female, psychologist; PhD)<br>CdJ (female; medical doctor, PhD)<br>SB (female; physiotherapist; Msc)<br>GB (male; physiotherapist; Msc)
2. Credentials	What were the researcher's credentials? E.g. PhD, MD	
3. Occupation	What was their occupation at the time of the study?	
4. Gender	Was the researcher male or female?	
5. Experience and training	What experience or training did the researcher have?	All the latter interviewers were new to qualitative research except for AF who already had experience with conducting interviews. AF helped during the preparation of the interview and provided support during the process of the interviews. All researchers were informed and trained on how he or she should conduct the interview during an investigator's meeting prior to the start of the trial. A written summary was provided to each coach after the meeting. During the same investigator's meeting, coaches were instructed on how to fill in the interview data (anonymized and transcribed verbatim) in the template excel file.
Relationship with participants		
6. Relationship established	Was a relationship established prior to study commencement?	No prior relationship between the interviewers and participants existed.
7. Participant knowledge of the interviewer	What did the participants know about the researcher? e.g.	Potential participants were informed and recruited in a standardized manner based on the summary part of the informed consent via

	personal goals, reasons for doing the research	1) phone calls 2) face-to-face contacts when participants were present at their respective center for their treatment or 3) via e-mail. In case participants had additional questions on the rationale of the trial, researchers were asked to refer to the informed consent where each part of the trial was explained in full detail. Potential participants were aware that the study was led by the PROactive consortium, which was funded by the Innovative Medicines Initiative (IMI).
8. Interviewer characteristics	What characteristics were reported about the inter viewer/facilitator? e.g. Bias, assumptions, reasons and interests in the research topic	All interviewers and researchers of the current trial formed part of the PROactive consortium, which had several work packages related to physical activity in patients with COPD. The aims, methodologies and statistical analyses of all work packages of the latter consortium were a priori defined to minimize bias. All participating centers and their respective researchers are well-known for their expertise in the field of physical activity in patients with chronic respiratory diseases.
**Domain 2: study design**		
Theoretical framework		
9. Methodological orientation and Theory	What methodological orientation was stated to underpin the study? e.g. grounded theory, discourse analysis, ethnography, phenomenology, content analysis	The analysis of the semi-structured interviews of the present trial was based on thematic analysis according to the six-steps framework as proposed by Braun and Clarke's [1].
Participant selection		
10. Sampling	How were participants selected? e.g. purposive, convenience, consecutive, snowball	Consecutive sampling was used to recruit participants for the MrPAPP trial in case they met the a priori defined inclusion criteria of the trial. In the present trial, participants of a wide spectrum of disease severity and a wide range of PA levels were included. Because of its liberal inclusion criteria, the present trial is likely to be relevant to patients seen in routine clinical practice. For the interviews itself, all participants who completed the MrPAPP trial (irrespective of whether this

		was successful or not) were invited during their final trial visit.
11. Method of approach	How were participants approached? e.g. face-to-face, telephone, mail, email	As previously mentioned, researchers approached and recruited participants in a standardized manner based on the summary part of the informed consent via 1) phone calls 2) face-to-face contacts when participants were present at each center for their treatment or 3) via e-mail for participation in the MrPAPP trial. For the interviews itself, all participants were invited face-to-face during the final visit of the MrPAPP trial.
12. Sample size	How many participants were in the study?	In total, 343 participants were recruited in the MrPAPP trial of which 171 were randomized to the intervention arm and 159 completed the trial. Interview data was collected from 145 participants. The sample size calculation of the MrPAPP trial was based on the primary outcome (i.e. physical activity) and is fully explained in the main paper of the MrPAPP trial [2]. No specific sample size calculation was performed for the interview data.
13. Non-participation	How many people refused to participate or dropped out? Reasons?	Twelve participants dropped out of the intervention group (of which 4 due to their inability to work with the smartphone application). Of the 159 participants that completed the trial, interview data was missing in 14 participants. Three participants refused to participate to the interview, as they were not satisfied with the intervention. In eleven participants, the interview data was missing because of lack of time.
Setting		
14. Setting of data collection	Where was the data collected? e.g. home, clinic, workplace	All the interview data collected during the MrPAPP trial was collected at the clinic or research department of each participating center (i.e. Sotiria General Hospital for Chest Diseases (Athens, Greece); ELEGI and COLT Laboratories, The Queen's Medical Research Institute, (Edinburgh, UK); Royal Brompton Hospital (London, UK); UZ Leuven Hospital (Leuven, Belgium); University Hospital Zurich (Zurich, Switzerland), Universitair Medisch Centrum Groningen (Groningen, the Netherlands).

15. Presence of non-participants	Was anyone else present besides the participants and researchers?	Interviewers were instructed to conduct the interview with the participants alone in a quiet room. In case a relative accompanied him/her to the center, they were asked to temporarily wait outside the room to minimize bias during the interviewing process.
16. Description of sample	What are the important characteristics of the sample? e.g. demographic data, date	Participants with COPD with a wide range of disease severity were included in the present trial. Data on gender, BMI, severity of airflow obstruction, exercise tolerance, quadriceps force and physical activity has been reported in the present manuscript (cfr. Table 1). Those participants from which we did not collect interview data (n=14) had similar characteristics (for any of the variables mentioned in the present manuscript) compared to the ones with interview data (n=145). In addition, each illustrative quote from the interview analysis was presented with the tertiles group of actual usage score and contact time of each participant together with his/her identification number.
Data collection		
17. Interview guide	Were questions, prompts, guides provided by the authors? Was it pilot tested?	The interview template has been provided in Multimedia Appendix 4. There was no pilot testing process conducted before the implementation of the interview.
18. Repeat interviews	Were repeat inter views carried out? If yes, how many?	Interviews were only conducted once with each participant.
19. Audio/visual recording	Did the research use audio or visual recording to collect the data?	No audio-recording was performed during all interviews.
20. Field notes	Were field notes made during and/or after the interview?	Interviewers were asked to note down all verbatim patient's answers during the interview.
21. Duration	What was the duration of the interviews?	Interviews were not timed and therefore no exact data on the duration of all interview was available. In the investigator's meeting after the trial ended, interviewers were asked after the trial to estimate how much time (on average) they needed to conduct the interview. According to the interviewers, it took roughly 15 minutes per patient to conduct the interview with a minimum and

		maximum of respectively 5 to 40 minutes.
22. Data saturation	Was data saturation discussed?	Despite the large sample size, we can not completely exclude whether data saturation was reached as we did not conduct another interview with the participants nor did we return transcripts to the respective participants.
23. Transcripts returned	Were transcripts returned to participants for comment and/or correction?	Transcripts were not returned to participants for comment and/or correction.
**Domain 3: analysis and findings**		
Data analysis		
24. Number of data coders	How many data coders coded the data?	HD & FR conducted the coding process according to the 6- step framework as proposed by Braun and Clarke's [1].
25. Description of the coding tree	Did authors provide a description of the coding tree?	The classification tree was used to create the table format (cfr. Table 2) in the results section.
26. Derivation of themes	Were themes identified in advance or derived from the data?	Thematic analysis was conducted inductively (i.e. themes emerged from the data, so without pre-determined coding frame) in excel. Themes identification was semantic.
27. Software	What software, if applicable, was used to manage the data?	Microsoft excel was used to collect, organize (via color coding) and analyze all interview data [3]. No other specialized software was used for the data coding, which was performed manually by HD & FR.
28. Participant checking	Did participants provide feedback on the findings?	Participants did not provide feedback on the findings, as the transcripts were not returned to them.
Reporting		
29. Quotations presented	Were participant quotations presented to illustrate the themes/findings? Was each quotation identified? e.g. participant number	Illustrative quotes were presented to illustrate the findings. A unique participant's number identified each quote and was complemented with the tertiles group of actual usage score and total contact time of each patient.
30. Data and findings consistent	Was there consistency between the data	The authors attempted to present the qualitative findings in the clearest way

	presented and the findings?	possible, in line with the verbatim findings and its accompanying codings. Findings were iteratively discussed between the researchers involved in the qualitative analysis process. Major and minor themes together with illustrative quotes were presented and described in the manuscript (Table 2).
31. Clarity of major themes	Were major themes clearly presented in the findings?	
32. Clarity of minor themes	Is there a description of diverse cases or discussion of minor themes?	

REFERENCES

1. Braun V, Clarke V. Using thematic analysis in psychology. Qual Res Psychol 2006; PMID:223135521
2. Demeyer H, Louvaris Z, Frei A, Rabinovich RA, De Jong C, Gimeno-Santos E, Loeckx M, Buttery SC, Rubio N, Van Der Molen T, Hopkinson NS, Vogiatzis I, Puhan MA, Garcia-Aymerich J, Polkey MI, Troosters T. Physical activity is increased by a 12-week semiautomated telecoaching programme in patients with COPD: A multicentre randomised controlled trial. Thorax 2017;72(5):415–423. PMID:28137918
3. Bree R, Gallagher G. Using Microsoft Excel to code and thematically analyse qualitative data: a simple, cost-effective approach. All Irel J Teach Learn High Educ 2016;
