# Supplementary material for: Smartphone-Based Physical Activity Telecoaching in Chronic Obstructive Pulmonary Disease: Mixed-Methods Study on Patient Experiences and Lessons for Implementation
Source: JMIR Mhealth Uhealth. 2018 Dec 21;6(12):e200. doi: 10.2196/mhealth.9774 (PMC6320438; doi:10.2196/mhealth.9774)
Supplement: Multimedia Appendix 7 [file mhealth_v6i12e200_app7.pdf]

**Multimedia Appendix 7: sensitivity analyses**

METHODOLOGY:

To confirm the findings from our tertiles approach, we have also conducted sensitivity analyses. In these sensitivity analyses, we aimed to investigate how both contact time and actual usage score as continuous variables were related to baseline demographic factors. Therefore, we conducted univariate regression models with respectively contact time and actual usage score as the outcome variable and the following variables as class variables; age, gender, body mass index (BMI), forced expiratory volume in 1 second % predicted ($FEV_1$ % predicted), six minute walking distance (6MWD), COPD assessment test (CAT), modified Medical Research Council score (mMRC), quadriceps force (QF) and PA.

Furthermore, we also aimed to analyze the association between 1) the actual usage by patients of the different components of the intervention and coach feasibility (i.e. contact time) with 2) the effectiveness of the intervention. Therefore, we conducted univariate and stepwise multivariate linear regression analyses (proc reg in SAS 9.4) to assess whether contact time and actual usage scores (as continuous variables) were, independently of covariates, related to change in steps per day. Change in PA was used as the outcome variable. Next to contact time and actual usage we used the following other class variables; age, gender, BMI, $FEV_1$, 6MWD, the number of acute exacerbations in the previous 12 months, mMRC-score, QF and PA [1]. Factors were entered in the stepwise multivariate regression model if they had a *P*-value <.20 in the univariate analysis.

RESULTS:

Univariate regression analysis revealed that higher contact time tended to be significantly associated with more severe airflow obstruction ($FEV_1$) and lower functional exercise tolerance (6MWD) (Multimedia Appendix 7, Table 1). This is in line with our tertiles approach, which showed that patients in the high contact time group had more severe airflow obstruction and tended to have a lower functional exercise tolerance compared to patients in the low contact time group.

**Multimedia Appendix 7, Table 1. Univariate regression analysis to assess the association between baseline demographic factors and the amount of contact time (in minutes). Data are presented as mean estimate (standard error).**

| Contact time in minutes | Mean estimate (SE[a]) | | *P*-value |
|---|---|---|---|
| Age in years | 1.01 | (0.73) | .13 |
| Gender (male) | -3.97 | (12.12) | .74 |
| BMI[b] in kg per $m^2$ | -0.82 | (1.08) | .45 |
| $FEV_1$[c] predicted percentage | -0.53 | (0.29) | .07 |
| 6MWD[d] in m | -0.09 | (0.06) | .09 |
| 6MWD predicted percentage | -0.32 | (0.36) | .28 |
| CAT[e] score | 0.48 | (0.78) | .54 |
| mMRC[f] score | 4.6 | (6.2) | .46 |
| QF[g] in kg | -0.76 | (0.49) | .14 |
| PA[h] in steps per day | -0.003 | (0.002) | .19 |

[a]SE: Standard Error.
[b]BMI: Body Mass Index.
[c]$FEV_1$: Forced Expiratory Volume in one second.
[d]6MWD: Six Minute Walk Distance; 6MWD was missing in 2 patients.
[e]CAT: COPD assessment test.
[f]mMRC: Modified Medical Research Council score for dyspnea.
[g]QF: Quadriceps Force ; QF was not measured in 2 centers and QF was missing in 27 patients.
[h]PA: Physical Activity; Valid PA measurements was present in 140 patients.

Multivariate regression analysis revealed that having more contact time was a significant independent predictor of a smaller improvement of PA after taking into account other factors (baseline 6MWD, $FEV_1$ % predicted, the number of acute exacerbations in the previous 12 months, mMRC-score and age) (Multimedia Appendix 7, Table 2). For having one minute more of contact time, patients had a mean estimate (standard error) change in PA of -9 (3) steps/day from 0 to 3 months (*P*-value=.004). This also confirmed our findings from the tertiles approach, in which patients who had more contact time with the coaches during the time of the study, had a significantly smaller increase in PA, after adjusting for covariates (*P* for trend=.01; cfr. Figure 5 in the manuscript).

**Multimedia Appendix 7, Table 2. Univariate and multivariate (stepwise) regression analysis to assess whether the amount of contact time (in minutes) and the total actual usage score (in percentages) are related to change in physical activity throughout the trial, independently of other factors. Data are presented as mean estimate (standard error). The adjusted $R^2$ of the final model was = 0.16; *P*<.0001. Two patients were excluded from the multivariate analysis due to missing six minute walking distance measurements (n=2).**

| | Univariate | | | Multivariate | | |
|---|---|---|---|---|---|---|
| **Δ[a] PA[b] in steps per day** | **Mean estimate(SE[c])** | | ***P*-value** | **Mean estimate (SE)** | | ***P*-value** |
| Age in years | 42 | (27) | .12 | 79 | (26) | **.003** |
| Gender (male) | 94 | (446) | .83 | | | |
| BMI[d] in kg per $m^2$ | -14 | (40) | .73 | | | |
| $FEV_1$[e] predicted percentage | 28 | (10) | **.008** | | | ns |
| 6MWD[f] in m | 6 | (2) | **.003** | 5 | (2) | **.02** |
| Number AE[g] last 12 months | -178 | (76) | **.026** | | | ns |
| mMRC[h] score | -662 | (222) | **.004** | -443 | (239) | **.07** |
| QF[f] in kg | 17 | (22) | .44 | | | |
| PA in steps per day | -0.08 | (0.08) | .31 | | | |
| Contact time in minutes | -9 | (3) | **.005** | -9 | (3) | **.004** |
| Actual usage in percentage | -0.56 | (9.2) | .95 | | | |

[a]Δ, change.
[b]PA: Physical Activity; Valid PA measurements was present in 140 patients.
[c]Standard Error.
[d]BMI: Body Mass Index.
[e]$FEV_1$: Forced Expiratory Volume in one second.
[f]6MWD: Six Minute Walk Distance; 6MWD was missing in 2 patients.

[g]AE, acute exacerbations.
[h]mMRC: Modified Medical Research Council score for dyspnea.
[f]QF: Quadriceps Force; QF was not measured in 2 centers and QF was missing in 27 patients.
[h]ns: non-significant.

In contrast to contact time, univariate regression analysis revealed no baseline demographic factors were related to a lower or higher total actual usage score (Multimedia Appendix 7, Table 3). This also confirms our tertiles approach in which we did not find any differences in patient characteristics when patients were divided into 3 groups according to their overall actual usage score (cfr. Table 4 in the manuscript).

**Multimedia Appendix 7, Table 3. Univariate regression analysis to assess predictors of actual usage score (in percentage). Data are presented as mean estimate (standard error). Twenty-nine patients had missing values for respectively QF measurements (n=27) and missing 6MWD (n=2).**

| **Actual usage in percentage** | **Mean estimate (SE[a])** | | ***P*-value** |
|---|---|---|---|
| Age in years | -0.04 | (0.25) | .87 |
| Gender (male) | 2 | (4) | .61 |
| BMI[b] in kg per $m^2$ | -0.63 | (0.36) | .12 |
| $FEV_1$[c] predicted percentage | -0.06 | (0.10) | .54 |
| 6MWD[d] in m | 0.01 | (0.02) | .51 |
| 6MWD predicted percentage | 0.03 | (0.12) | .79 |
| CAT[e] score | 0.09 | (0.26) | .73 |
| mMRC[f]-score | -0.96 | (2.11) | .65 |
| QF[h] in kg | 0.04 | (0.05) | .40 |
| PA[g] in steps per day | 0.0006 | (0.0007) | .43 |

[a]Standard Error.
[b]BMI: Body Mass Index.
[c]$FEV_1$: Forced Expiratory Volume in one second.
[d]6MWD: Six Minute Walk Distance; 6MWD was missing in 2 patients.
[e]CAT: COPD assessment test.

[f]mMRC: Modified Medical Research Council score for dyspnea.
[h]QF: Quadriceps Force; QF was not measured in 2 centers and QF was missing in 27 patients.
[g]PA: Physical Activity; valid PA measurements was present in 140 patients.

Furthermore, regression analyses revealed that there was no significant association between total actual usage score and change in PA from 0 to 3 months (*P*-value=.95; Multimedia Appendix 7, Table 2). This is also in line with findings from our tertiles approach (cfr. Figure 6 in the manuscript).

REFERENCES

1. Demeyer H, Louvaris Z, Frei A, Rabinovich RA, De Jong C, Gimeno-Santos E, Loeckx M, Buttery SC, Rubio N, Van Der Molen T, Hopkinson NS, Vogiatzis I, Puhan MA, Garcia-Aymerich J, Polkey MI, Troosters T. Physical activity is increased by a 12-week semiautomated telecoaching programme in patients with COPD: A multicentre randomised controlled trial. *Thorax* 2017; 72: 415–423.
