# Supplementary material for: Smartphone-Based Physical Activity Telecoaching in Chronic Obstructive Pulmonary Disease: Mixed-Methods Study on Patient Experiences and Lessons for Implementation
Source: JMIR Mhealth Uhealth. 2018 Dec 21;6(12):e200. doi: 10.2196/mhealth.9774 (PMC6320438; doi:10.2196/mhealth.9774)
Supplement: Multimedia Appendix 8 [file mhealth_v6i12e200_app8.pdf]

Illustrative quotes on findings of the thematic analysis of the interview data. These are categorized under 1) technical aspects and 2) aspects related to the content of the intervention.

Technical aspects

1. Positive experiences
   - No technical problems: *"I had no technical difficulties with any of the devices of the intervention.* [Patient 64, high actual usage score, low contact time]

   - Working with app: *"I was quickly used to working with the smartphone. The step counter was also easy to use."* [Patient 34, high actual usage score, medium contact time]

2. Issues/problems
   - Help from others: "*When I experienced issues with the phone, I called S. and she helped me to solve the problem*." [Patient 112, medium actual usage score, high contact time]

   - Speed of interaction with the app: *"The smartphone was often slow. I often needed to wait 10 to 15 minutes before I could send my steps in the evening*." [Patient 119, low actual usage score, medium contact time]

   - App problems: *"I was not always able to send my steps due to Bluetooth connection problems or unstable internet connection.*" [Patient 85, medium actual usage score, low contact time]

   - Step counter: "*My cycling activities were not well captured by the step counter*." [Patient 25, low actual usage, medium contact time]

Aspects related to the content of the intervention

1. Positive experiences
   - Step counter: *"The step counter was the most useful part and I was a lot more compliant with this than completing the home exercises*." [Patient 6, high actual usage score, low contact time]

   - Graphs: *"I particularly liked the graph at end of the day displaying achievements*." [Patient 102, medium actual usage score, low contact time]

- Nice experience: *"The step counter and application were very helpful to increase my outdoor activities*." [Patient 111, high actual usage score, low contact time]

- Being monitored: "*The feeling of being monitored motivated me to be more active*." [Patient 20, high actual usage score, medium contact time]

- Family participation: *"Even my wife bought a step counter so we could reach the goal together*." [Patient 85, low actual usage, medium contact time]

2. Issues/problems
    - Goals: *"The goal was too high at the end of study. I felt under pressure."* [Patient 63, high actual usage, high contact time]

    - Variation: "*The tasks on the application lacked variation. I did not read the morning messages every day."* [Patient 141, low actual usage, medium contact time]

    - Barriers: "*The goals were not adapted to the weather and to the time period of the year (e.g. on Christmas).*" [Patient 78, medium actual usage score, high contact time]

    - Motivational issues: "*I wouldn't use the intervention again. It did not motivate me to be more active.*" [Patient 14, low actual usage score, high contact time]

3. Outcome

    - New routine: "*Brilliant, I increased my daily steps by walking more outdoors, leaving the car, doing more households and waking up earlier*." [Patient 54, high actual usage score, low contact time]
