# Supplementary material for: Smartphone-Based Physical Activity Telecoaching in Chronic Obstructive Pulmonary Disease: Mixed-Methods Study on Patient Experiences and Lessons for Implementation
Source: JMIR Mhealth Uhealth. 2018 Dec 21;6(12):e200. doi: 10.2196/mhealth.9774 (PMC6320438; doi:10.2196/mhealth.9774)
Supplement: Multimedia Appendix 9 [file mhealth_v6i12e200_app9.pdf]

Multimedia Appendix 9, Table 1. Actual usage of the different intervention tasks according to gender. Data are expressed as median (25th and 75th percentiles), unless stated otherwise.

Variables	Female (n[a]=58)	Male (n=101)	*P*-value
Wearing step counter (days per week)	6.4 (5.9-6.6)	6.6 (6.0-6.8)	.22
Send activity (days per week)	3.8 (2.3-5.3)	4.3 (2.4-5.8)	.27
Daily goal (days per week)	3.9 (2.0-5.5)	4.2 (2.4-6.0)	.27
Daily feedback (days per week)	1.9 (0.7-4.0)	2.3 (0.8-4.1)	.53
Weekly feedback (%[b])	50 (22-70)	63 (30-80)	.31

[a]number of patients

[b]percentage

Multimedia Appendix 9, Table 2. Actual usage of the different intervention tasks according to age (patients <65 years vs patients ≥65 years). Data are expressed as median (25th and 75th percentiles), unless stated otherwise.

Variables	<65 years (n[a]=67)	≥65 years (n=92)	*P*-value
Wearing step counter (days per week)	6.4 (6.1-6.8)	6.3 (5.8-6.9)	.71
Send activity (days per week)	4.2 (2.0-5.6)	4.1 (2.4-5.5)	.92
Daily goal (days per week)	3.4 (1.9-5.9)	4.4 (2.4-5.9)	.19
Daily feedback (days per week)	2.4 (0.7-4.2)	2.1 (0.9-3.8)	.68
Weekly feedback (%[b])	50 (18-80)	58 (36-75)	.44

[a]number of patients

[b]percentage
