# Supplementary figures and images for: Smartphone-Based Physical Activity Telecoaching in Chronic Obstructive Pulmonary Disease: Mixed-Methods Study on Patient Experiences and Lessons for Implementation
Source: JMIR Mhealth Uhealth. 2018 Dec 21;6(12):e200. doi: 10.2196/mhealth.9774 (PMC6320438; doi:10.2196/mhealth.9774)

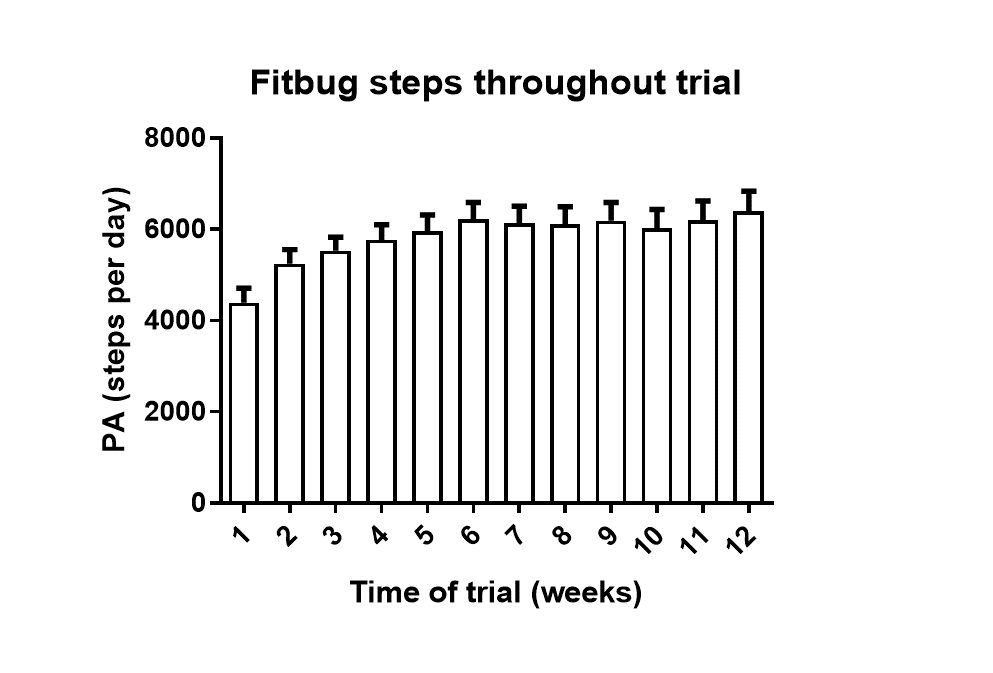

Supplement: Multimedia Appendix 10 [file mhealth_v6i12e200_app10.png]
